# A lab-on-a-chip approach integrating in-situ characterization and reactive transport modelling diagnostics to unravel (Ba,Sr)SO_4_ oscillatory zoning

**DOI:** 10.1038/s41598-021-02840-9

**Published:** 2021-12-08

**Authors:** Jenna Poonoosamy, Mohamed Mahrous, Enzo Curti, Dirk Bosbach, Guido Deissmann, Sergey V. Churakov, Thorsten Geisler, Nikolaos Prasianakis

**Affiliations:** 1 Institute of Energy and Climate Research (IEK-6): Nuclear Waste Management and Reactor Safety, Forschungszentrum Jülich GmbH, 52425 Jülich, Germany; 2 Laboratory for Waste Management, Paul Scherrer Institut, 5232 Villigen PSI, Switzerland; 3 Institute of Geological Sciences, University of Bern, 3012 Bern, Switzerland; 4 Institut Für Geowissenschaften, Rheinische Friedrich-Wilhelms-Universität Bonn, Bonn, Germany

**Keywords:** Mineralogy, Geochemistry, Pollution remediation

## Abstract

The co-precipitation of sulphate minerals such as celestine and barite is widely studied because their formation is ubiquitous in natural and anthropogenic systems. Co-precipitation in porous media results in crystallization of solid solutions yielding characteristics such as oscillatory zoning that are rarely observed in bulk solution or in batch experiments. In the past, the precipitation of compositionally-zoned (Ba,Sr)SO_4_ crystals was observed post-mortem in macroscopic silica gel counter-diffusion experiments. Their formation was originally explained by the difference in the solubility products of the end-members combined with diffusion-limited transport of solutes to the mineral-fluid interface, while a later study favored the idea of kinetically controlled reactions. With recent advances combining in-operando microfluidic experiments and reactive transport modelling, it is now possible to verify hypotheses on the driving forces of transport-coupled geochemical processes. We developed a “lab on a chip” experiment that enabled the systematic study of the nucleation and growth of oscillatory-zoned (Ba,Sr)SO_4_ crystals in a microfluidic reactor. The compositions of the solid solutions were determined by in-situ Raman spectroscopy. Our investigation shows (1) that the composition of the nucleating phases can be approximated using classical nucleation theory, (2) that the oscillatory zoning is not solely controlled by the limited diffusional transport of solutes, and (3) that nucleation kinetics plays a major role in the switch between different stoichiometric compositions. The zoning phenomena is governed by the complex interplay between the diffusion of reactants and the crystallization kinetics as well as other factors, e.g. surface tension and lattice mismatch.

## Introduction

Solid solutions including barium sulphate (BaSO_4_) as a major component are widely present in various natural and anthropogenic systems. They are commonly formed during hydraulic fracturing in geothermal systems for energy production^1^, uranium mining tailings^2^ and treatment of industrial waste and contaminated water^3–6^. The low solubility of BaSO_4_ enables the removal of structure-compatible trace contaminants and radionuclides such as ^226^Ra and ^90^Sr. In geological repositories for nuclear waste, co-precipitation with BaSO_4_ solid solutions is expected to reduce the solubility of ^226^Ra, a radionuclide continuously produced by radioactive decay in uranium-bearing waste streams^7–10^, thus limiting its migration towards the biosphere. The high relevance of BaSO_4_ containing solid solutions as a sink of ^226^Ra and ^90^Sr has triggered the development of advanced thermodynamic models that allow to reliably assess the solubility of these radionuclides^11,12^. However, such models are not sufficient to describe the fate of such contaminants in subsurface systems. This is because co-precipitation processes in the porous matrices of the subsurface are typically influenced by the complex interplay of solute transport and dissolution/precipitation kinetics^8^. The relatively slow advective velocities and thus diffusion-dominated transport of solutes encountered in various settings in the in the subsurface (e.g. in the engineered barrier system of nuclear waste repositories or in tight rock formations) will induce specific effects during the crystallization of solid solutions which are not or only rarely observed in bulk solution/batch experiments. One such phenomenon is the oscillatory zoning, i.e. the formation of successive layers of minerals with different composition or properties.

Oscillatory zoning is common for binary solid solutions where the solubility products of the end members differ by several orders of magnitude, e.g. Ba(SO_4_, CrO_4_) and (Ba,Sr)SO_4_^13–15^. Previously, (Ba,Sr)SO_4_ oscillatory zoning (i.e. successive layers of respectively Sr and Ba enriched solid solutions) was observed in counter-diffusion experiments^16^. The experimental setup consisted of a porous matrix of silica gel placed between two columns filled with a mixed solution of barium chloride (BaCl_2_) and of strontium chloride (SrCl_2_) solution on one side, and a sodium sulphate (Na_2_SO_4)_ solution on the opposite side. The slow diffusion of solutes through the porous gel triggered the crystallization of zoned (Ba,Sr)SO_4_ crystals. This phenomenon was explained by the alternate consumption of barium (Ba) and strontium (Sr) in the pore solution in contact with the crystallized Ba and Sr enriched surface layers of the solid solutions, respectively, in a system characterized by slow diffusion of solutes. In principle, the precipitation of the lower solubility end-member (in this case BaSO_4_) was expected to occur first. Nevertheless, the authors also reported a strontium enriched solid solution as first precipitate. They suggested that the nucleation took place under non-equilibrium conditions^15^ with widely different threshold supersaturations for the two components (around 10,000 and 100 for BaSO_4_ and SrSO_4_, respectively) that had to be reached for nucleation to occur in the porous gel. The lower threshold for SrSO_4_ was reached faster and therefore a Sr-enriched solid solution nucleated first. Later, Pina et al.^17^ proposed a “simplified” model based on classical nucleation theory (CNT) to predict the composition of the nucleating phase, but such an approach to predict the composition of the nucleating phase still needs to be verified experimentally. Additional theoretical approaches to describe the phenomenon include cellular automata where Ba and Sr follow a set of rules for their motion and attachment to the crystal surfaces^18^. Such a model implies that the composition of the precipitating phase depends on the solution chemistry and the substrate.

Understanding the mechanisms that drive oscillatory zoning will enable to build realistic conceptual approaches that describe solid solution precipitation and therefore the fate of mobile radionuclides like ^226^Ra in the subsurface. In this work, we revisit the oscillatory zoning of (Ba,Sr)SO_4_ solid solutions by performing new experiments using a novel micronized lab-on-a-chip device^19^. The device allows carrying out counter diffusion experiments with real-time monitoring of mineral growth by time lapse optical microscopy and in-situ characterization by micro-Raman spectroscopy. The experiments were complemented by reactive transport models, which allow predicting the time–space evolution of transport pathways, aqueous solute concentrations and relevant thermodynamic/kinetic parameters (e.g., saturation indices, precipitation rates). The models are based on lattice Boltzmann methods^20,21^ for the evaluation of the initial stoichiometric saturation ratios in the system and a continuum scale approach to resolve the solute transport coupled to co-precipitation processes^22,23^ and are able to simulate different scenarios that shed light on the key mechanisms responsible for the observed (Ba,Sr)SO_4_ zoning phenomena. In this study, we performed microfluidic experiments and applied and evaluated theoretical approaches (stoichiometric supersaturation function, the delta function^15^ as well a classical nucleation theory extended to solid solutions^24^) to predict the composition of the nucleating phase. This enabled us to assess whether the crystal growth zonation is controlled by diffusion of solutes to the mineral–water interface or by crystallization kinetics.

## Methods

### Experimental setup and procedure

**Figure 1:**
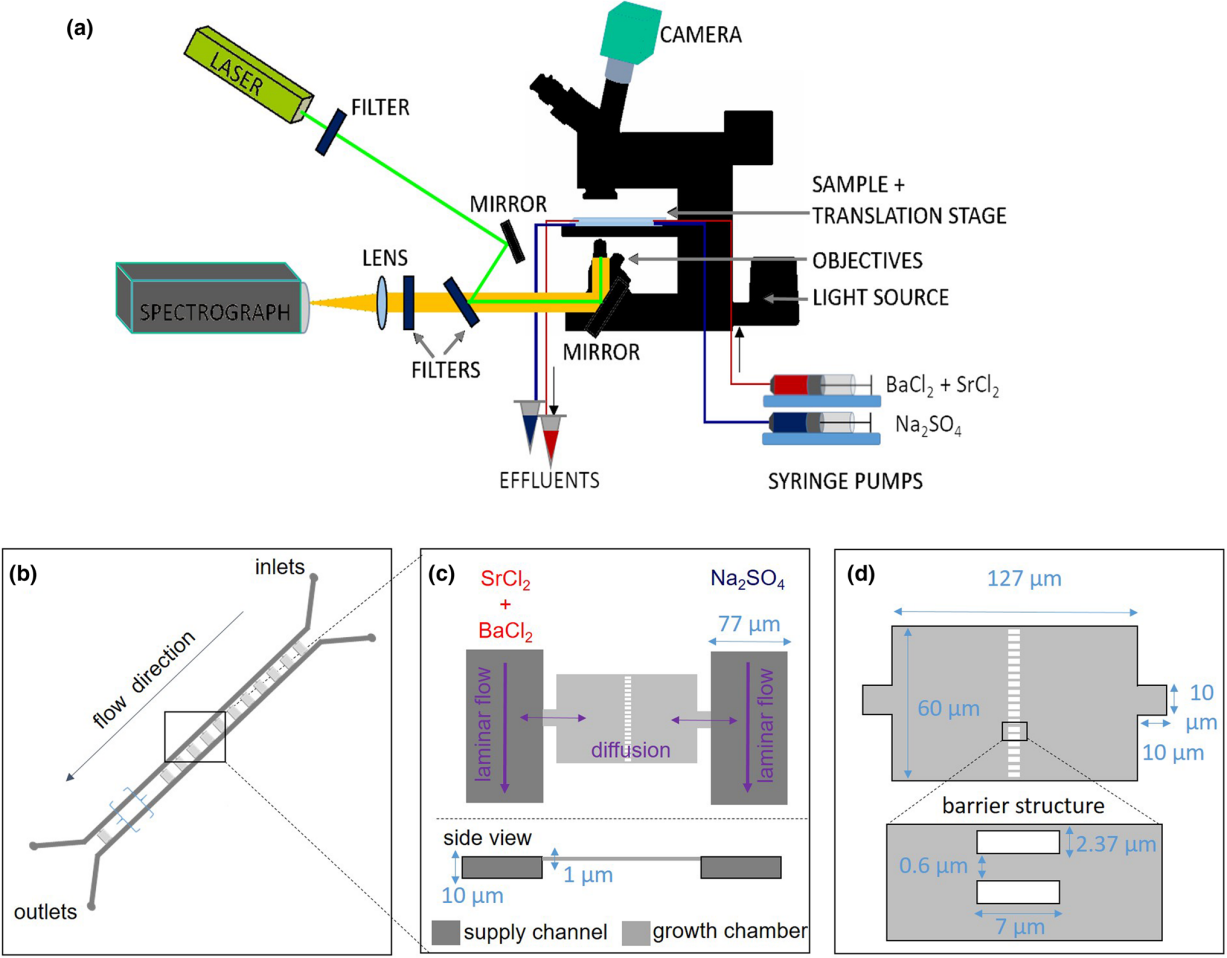
(**a**) Schematic representation of the microfluidic setup, (**b**) microfluidic reactor with an array of 50 growth chambers, (**c**) top view of microfluidic growth chamber with the two adjacent supply channels, (**d**) an enlargement of the growth chamber with an array of pillars constituting a barrier structure in the middle.

The experimental setup consists of a microfluidic reactor that is connected to pumps and monitored by optical microscopy and Raman spectroscopy (Fig. 1a). The microfluidic reactor is composed of two adjacent supply channels and 50 growth chambers (Fig. 1b). The growth chambers have a length of 127 μm and 60 μm width. A narrow channel of 10 μm by 10 μm connects the supply channels to the growth chambers. The narrow connections between the supply channels and the growth chambers enable a diffusion dominated transport regime in the growth chamber. The barrier structures (Fig. 1d) consist of an array of rectangular pillars (of 7 μm length and 2.37 μm width) and distanced by 0.6 μm placed in the middle of the chamber. They maintain mechanical stability of the chamber and serve as substrate to initiate the nucleation process^22^. The microfluidic reactor was made out of PDMS (Polydimethylsiloxane) and closed with a glass cover.

The two inlets were each connected to a 1 mL syringe, dispensing a mixed solution of 9.9 mM of SrCl_2_ and 1 mM BaCl_2_ and a solution of 10.9 mM Na_2_SO_4_, respectively (Fig. 1c). The two outlets were linked to two effluent vessels. The microfluidic reactor was initially filled with deionized water, followed by the injection of the reacting solutions at a rate of 500 nL min^−1^ using a syringe pump (Nemesys, Cetoni GmBH, Germany) for 20 h. The diffusion of the reacting solutions fostered the precipitation of (Ba,Sr)SO_4_ solid solutions in the chambers of the microfluidic device. The experiment was conducted at ambient temperature (21 °C) and pressure.

The microfluidic experiment was monitored using an automated inverted microscope (Witec alpha300 Ri Inverted Confocal Raman Microscope, which consists of an inverted Nikon Ti-2 U as base microscope) with a Nikon 100× oil immersion objective, having a numerical aperture (NA) of 1.25, a working distance of 0.23 mm, and a cover glass correction. The instrument is equipped with 70 mW Nd:YAG laser (λ = 532 nm) and a thermoelectrically cooled charge-coupled device (CCD). During the experiment, optical microscopy images of the first 5 chambers were recorded periodically. Time-lapse snapshots of crystal growth were analysed following the approach of Poonoosamy et al.^22^.The images were segmented by colour threshold values chosen manually, based on visual identification of the crystal threshold method in the ImageJ software. The thresholding consisted of segmenting pixels into foreground and background, which resulted in a binary image with the foreground pixels that had a value of one (the solid solution precipitates) and background pixels that had a value of zero. The area with precipitates enabled the calculation of the volume and therefore molar amounts of precipitates. This continuous monitoring allowed determining the apparent initial precipitation rates. After 20 h of injection, hyperspectral Raman images of the chambers over the region of interest were recorded with a step size of 400 nm by continuous x–y stage movement. Raman intensities were recorded for 0.1 s in the wavenumber range from 300 to 1300 cm^−1^. The laser power was set to 50 mW and a grating with 1800 grooves/mm was chosen. With this setup, the spectral resolution was 2 cm^−1^. The theoretical, diffraction-limited lateral and axial resolutions of the Raman measurements at the sample surface were calculated at ~ 520 nm and ~ 2043 nm using Eq. (3) in 4 in Everall^25^, and considering the refraction index of the immersion medium (n = 1.55).

In addition, the Raman spectra of synthetic BaSO_4_ (99.99% from Chempur), SrSO_4_ (99.99% from Chempur) and the cured PDMS were collected for 0.4 s in the wavenumber range from 200 to 1400 cm^−1^. These measurements served as standards for further evaluation of our experimental data. The free sulphate ions (SO_4_^−2^) have characteristic ν_1_ and ν_3_ bands corresponding to the symmetric and the anti-symmetric stretching modes, respectively, and ν_2_ and ν_4_ bands corresponding to the bending vibrations. The intense ν_1_(SO_4_) band for BaSO_4_ and SrSO_4_ are located at 988 and 1001 cm^−1^, respectively, and thus do not overlap with bands from PDMS. The stoichiometric composition of the solid solutions was determined using the True match module (Witec Control 5.6 Software), which uses an algorithm based on least square fitting to determine the mole percentage of each end-member. In addition, we applied a different methodology based on Vegard’s law behaviour observed for sulphate solid solutions, i.e., lattice parameters as well as vibrational frequencies are linearly correlated with composition^26^. This method is based on a linear interpolation of the positions (cm^−1^) of the ν_1_(SO_4_) band maxima as function of the mole fraction of both end members. Both methods gave consistent results and were in agreement with measured Raman spectra of (Ba,Sr)SO_4_ solid solutions standards measured in this work (see supplement S8) and in the literature^27^.

### Theoretical approaches

We tested and evaluated well-established theoretical approaches to predict the composition of the nucleating phase based on (1) thermodynamic equilibrium and (2) kinetics. The composition of the thermodynamically most stable solid solution can be determined from the composition of the aqueous solution in a consistent manner either by determining the maximum of the stoichiometric supersaturation function (Ω_st_)^15^ or the crossing point of the two delta functions δB and δC^28^ (see also supplement S1 and S6). The stoichiometric supersaturation function (Ω_st_) for solid solutions was computed for the entire compositional range from X_Ba_ = 0 to X_Ba_ = 1 using the equation below^15^:

1 $$\Omega _{st} \left( {X_{Ba} } \right) = \frac{{\left( {a_{{Ba^{2 + } }} } \right)^{{X_{Ba} }} \left( {a_{{Sr^{2 + } }} } \right)^{{X_{Sr} }} \left( {a_{{SO_{4}^{2 - } }} } \right)}}{{\left( {K_{{BaSO_{4} }} \gamma_{{BaSO_{4} }} X_{Ba} } \right)^{{X_{Ba} }} \cdot \left( {K_{{SrSO_{4} }} \gamma_{{SrSO_{4} }} X_{Sr} } \right)^{{X_{Sr} }} }}$$

where $a_{{Ba^{2 + } }}$, $a_{{Sr^{2 + } }}$ and $a_{{SO_{4}^{2 - } }}$ represent the free ion activities in the aqueous solution considering the extended Debye–Huckel ionic strength activity model; $K_{{BaSO_{4} }}$ and $K_{{SrSO_{4} }}$, the solubility products of the end-members BaSO_4_ and SrSO_4_ equal to 10^−9.97^ and 10^−6.63^ mol^2^ L^−2^ respectively at 298.15 K^29^; and $X_{Ba}$ and $X_{Sr}$, the molar fractions of BaSO_4_ and SrSO_4_ in the solid. $\gamma_{{BaSO_{4} }}$ and $\gamma_{{SrSO_{4} }}$ are the activity coefficients of the end-members in the solid solution based on the Thompson-Waldbaum model^30^ and assuming a regular mixing model with a Margules interaction parameter, *w*, of 4950 J mol^−1^^11^. Any solid solution with a stoichiometric saturation $\Omega _{st} > 1$ can potentially precipitate while those with a $\Omega _{st} < 1$ will dissolve. The mole fractions corresponding to the maximum of this function gives the thermodynamically most stable solid solution for a given aqueous solution composition.

Solid solution nucleation kinetics is treated in the framework of classical nucleation theory^24^. The nucleation rate $J\left( {X_{Ba} } \right)$ for a given solid solution composition *X*_Ba_ can be expressed as:

2 $$J\left( {X_{Ba} } \right) =\Gamma _{{\left( {X_{Ba} } \right)}} exp\left( { - \frac{{\Delta G_{{c\left( {X_{Ba} } \right)}} }}{kT}} \right)$$

where *k* is the Boltzmann constant, *T* is the absolute temperature (298.15 K), Γ a pre-exponential factor related to the solubility and ΔG_c_ in the energy required for the formation of a nucleus of critical size. ΔG_c_ is given as:

3 $$\Delta G_{{c\left( {X_{Ba} } \right)}} = \frac{{\beta v_{{\left( {X_{Ba} } \right)}}^{2} \sigma_{{\left( {X_{Ba} } \right)}}^{3} }}{{\left( {kTln\Omega _{{st\left( {X_{Ba} } \right)}} } \right)^{2} }}$$

where $v_{{\left( {X_{Ba} } \right)}}$ is the molecular volume of the smallest building unit of the nuclei (“monomer”), *β* is a geometry factor that depends on the shape of the nucleus and was set to 16.8 (value for spheres)^31^, and $\sigma_{{\left( {X_{Ba} } \right)}}$ (J m^−2^) is the effective specific surface energy of the cluster/solution interface and is a linear function of the solid solution composition. The effective specific surface energy for pure SrSO_4_ and BaSO_4_ is computed following Eq. 10 of Poonoosamy et al.^22^, considering a contact angle of 65° for SrSO_4_ nucleation on PDMS^22^ and between 21° to 45° for the nucleation of a sulphate phase on a sulphate phase (calculated from the effective σ of the nucleation of barite on barite^32^ and barite on celestine 33). The surface tension, σ, of the cluster/solution interface used for pure barite and celestine are 0.134 and 0.092 J m^−2^ respectively^31^. The “monomer” volume was computed by estimating the molecular volumes of the aqueous BaSO_4_(aq) and SrSO_4_(aq) complexes from the ionic radii of Ba^2+^, Sr^2+^, S^6+^ and O_2_^−^ and was considered to be a linear function of the solid solution composition with 8.6 × 10^−29^ m^3^ and 8.21 × 10^−29^ m^3^ for BaSO_4_(aq) (pure barite) and SrSO_4_(aq) (pure celestine), respectively.

The pre-exponential factor, Γ, is given as:

4 $$\Gamma _{{\left( {X_{Ba} } \right)}} = 2\pi Z_{{X_{Ba} }} DN_{0} N_{1} d_{{cX_{Ba} }}$$

where *D* is the diffusion coefficient of BaSO_4_ (aq) and SrSO_4_(aq) monomers, which was set to 9.3 × 10^−10^ m^2^ s^−1^^34^, and *d*_c_ the diameter of the critical nucleus, $d_{c} = \frac{{4\sigma_{{\left( {X_{Ba} } \right)}} v_{{\left( {X_{Ba} } \right)}} }}{kTln\Omega }$. N_1_ and N_0_ are the number of monomers per unit volume of fluid and the number of nucleation sites, respectively. N_0_ was set to 2.5 × 10^13^ m^−3^, the same value used by Prieto^34^ for barite. N_1_ depends on supersaturation and was evaluated by geochemical modelling using GEMS (https://gems.web.psi.ch/) as the sum of SrSO_4_ and BaSO_4_ monomers. Z is the Zeldovich factor given as:

5 $$Z_{XBa} = \sqrt {\left( {\frac{{\Delta G_{{c\left( {X_{Ba} } \right)}} }}{{3\pi kT\left( {n_{{cX_{Ba} }} } \right)^{2} }}} \right)}$$

with the number of monomers in the critical nucleus, n_c_, given as:

6 $$n_{cXBa} = \left( {\frac{{2\sigma_{{\left( {X_{Ba} } \right)}} a_{{X_{Ba} }} }}{{3kTln\Omega _{{st(X_{Ba} )}} }}} \right)^{3}$$

where $a_{{X_{Ba} }}$ is a linear interpolation of the area occupied by a molecule over the compostion, with *a* equal to 9.42 × 10^−19^ and 9.14 × 10^−19^ m^2^ for barite and celestine, respectively.

The crystal growth rate of (Ba,Sr)SO_4_ was evaluated from the experimental datasets of Weber et al.^14^ (see supplement S1 for further details). The precipitation rate r_(XBa)_ [mol s^−1^] for a given stoichiometric composition of (Sr,Ba)SO_4_ follows a second order reaction (similar to pure barite^35^), the kinetic constant, *k*_XBa_, of a given stoichiometry can be calculated using the relationship:

7 $$r_{{\left( {XBa} \right)}} = S_{s} k_{XBa} \left( {1 -\Omega _{{st\left( {XBa} \right)}} } \right)^{2}$$

where *S*_s_ is the reactive surface area [m^2^], *k*_XBa_ is the kinetic constant and evaluated as a linear function of the kinetic constants of the end members with *k* (SrSO_4_) and *k* (BaSO_4_) equal to 4.8 × 10^−9^^36,37^ and 1.5 × 10^−11^ mol m^−2^ s^−1^^35^, respectively.

The crystallization rate, r_crys(XBa)_ (mol s^−1^) is defined as the sum of the nucleation rate and crystal growth rate and is given by Eq. (8):

8 $$r_{{crys\left( {XBa} \right)}} = \frac{{J\left( {X_{Ba} } \right)Vn_{cXBa} }}{NA} + r_{{\left( {XBa} \right)}}$$

where *V* is the volume in m^3^ where nucleation can occur, here equal to the volume of the growth chamber and NA is Avogadro’s number (6.02 × 10^23^ mol^−1^).

### Numerical modelling and diagnostics, towards digital twins

For the numerical modelling, several numerical algorithms are used at different steps in order to augment the information that can be extracted from the experiments as well as for the determination of:

1. The velocity field
  The 3D flow field in the growth chamber was simulated with computational fluid dynamics using the software COMSOL Multiphysics 5.3a (COMSOL AB, Stockholm, Sweden see supplement S2); this software was also used to provide a first estimate of solute concentration distributions.
2. The concentration gradients of the different components, the corresponding geochemical speciation and the saturation indices with respect to the observed precipitates during the initial phase of the experiment
  2D lattice Boltzmann pore level simulations^38^ were conducted (see references in supplemental material S4: 1, 3) for the calculation of the transient initial mixing of the solutes in order to resolve the aqueous concentrations of the species within the experimental chambers. The geochemical solver was coupled in a form of a surrogate model, which was trained using machine learning similar to (see references in S4: 2, 3). This provided several orders of magnitude faster calculations compared to the time that would be needed if a geochemical speciation solver would have been coupled directly to the lattice Boltzmann solver. The transient saturation index SI=log Ω with respect to the each precipitating phase was calculated until the chemical system reached a mixing steady state, describing the conditions before the first crystallization event occurred (see supplement S4 for more details).
3. The transport induced solid solution precipitation/evolution of precipitation

1D Open-GeoSys-GEMs (OGS-GEMS^39,40^) at the continuum level were conducted to decipher whether the zoning phenomena is a consequence of a diffusion-controlled precipitation or of a kinetically controlled reaction. We conducted two reactive transport studies at the continuum scale: (1) study 1, considering an instantaneous precipitation (thermodynamic equilibrium) of the two predominant solid solution compositions as extracted from the experiments, and (2) study 2 considering the kinetically controlled precipitation. In the continuum model, we projected the experimental setup to a 1D computational domain of 127 µm length and of 60 µm^2^ cross-sectional area, setting up a counter diffusion simulation with the same concentration boundary conditions as in the experiments (see supplement S3 for details). The 1D geometry was discretized into a grid composed of elements of 5 µm length as well as with a finer mesh discretization composed of finite elements of 1 µm length. The advection-diffusion reaction equation (ADRE) (equations S3.1–2 in S3) was solved using the OGS-GEMS code. For the case study 2, the precipitation rate of the stoichiometric composition was accounted for by crystal growth kinetics only (Eq. 7).

## Results

### Crystal growth and compositional zonation

**Figure 2:**
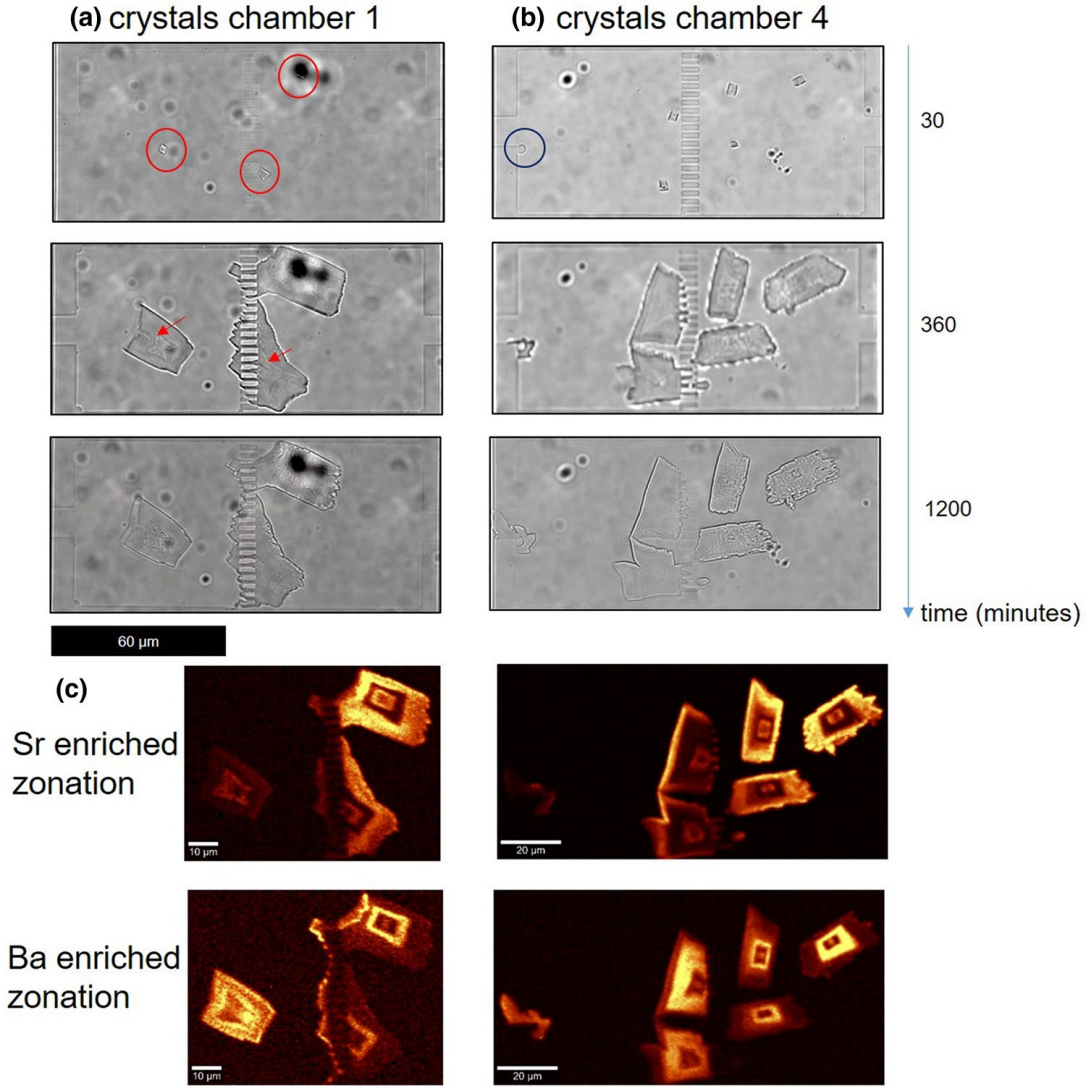
Temporal evolution of crystals in chamber 1 (**a**) and 4 (**b**) as revealed by optical microscopy. Image (**c**) shows hyperspectral Raman images of the ν_1_ (SO_4_) intensities from crystals formed after 1200 min in chamber 1 and 4 with intensities of 1000 ± 3 and 994 ± 3 cm^−1^.

The ingress of the mixed solutions of barium and strontium chloride solutions and sodium sulphate into the microfluidic reaction chambers triggered the formation of euhedral shaped crystals. The crystals were clearly distinguishable after 30 min reaction time (red circles in Fig. 2a) in the monitored chambers. These crystals occurred at the pillars or at regions of irregularities on the wall of the chambers (blue circle in Fig. 2b). The crystals grew continuously for 400 min, at which time complete clogging of the mass transport pathways was observed, either due to the complete obstruction of the pillars or because of the formation of new crystals at the entrance of the respective growth chamber, preventing further mass exchange between the supply channels and the growth chambers. The crystals appeared to grow by individual layers with alternating Sr-rich and Ba-rich composition, a zonation phenomenon whose boundaries can be observed already by optical microscopy (red arrows in Fig. 2a). The respective composition was resolved by Raman imaging (Fig. 2c) showing the distribution of strontium and barium enriched solid solutions within the single crystals. After the clogging event at the pillars, there was no further exchange of solutes between the two supply channels, therefore the aqueous solution becomes undersaturated with respect to the solids and the crystals dissolve between 600 and 1200 min.

**Figure 3:**
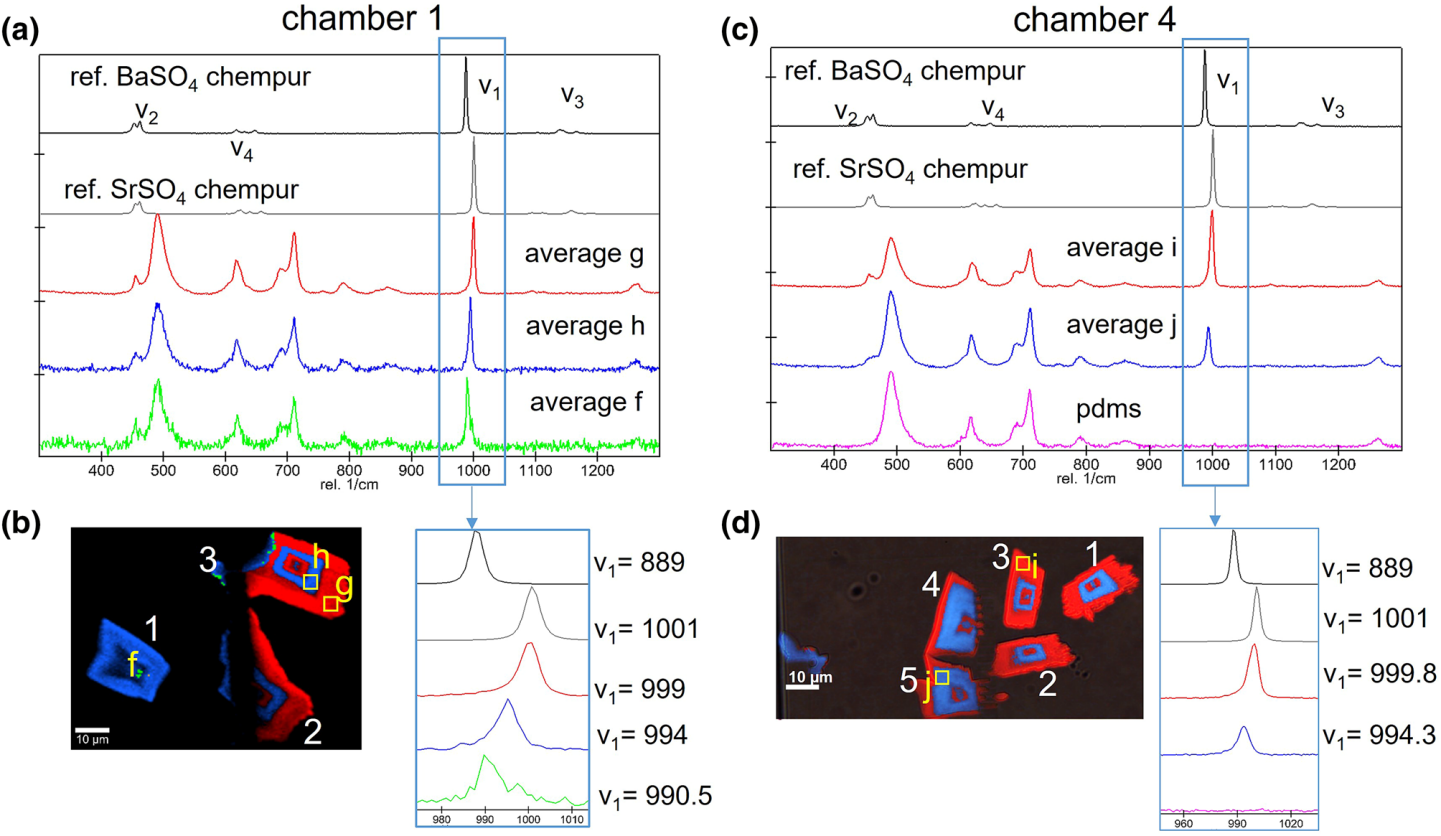
(**a**) Comparison of the average Raman spectra of the barium (**f**, **h**) and strontium (**g**) enriched layers of selected crystals in chamber 1 and those of commercial 99% pure BaSO_4_ and SrSO_4_, (**b**) locations where the spectral averaging was done in chamber 1 with an enlargement of the ν_1_(SO_4_) band, (**c**) comparison of the average Raman spectra of the barium (**j**) and strontium (**i**) enriched layers of selected crystals in chamber 4 and those of commercial 99% pure BaSO_4_ and SrSO_4_ and PDMS, (**d**) locations where the spectral averaging was done in chamber 4 along with corresponding Raman spectra of the ν_1_(SO_4_) frequency region.

The average Raman spectra of the individual layers were calculated for all crystals in the reaction chambers. Figure 3 shows only those of crystals in chamber 1 and 4 as typical examples. The ν_1_(SO_4_) frequency of all measured spots of the crystals is distinct and intermediate between those of pure BaSO_4_ and SrSO_4_, indicating without any doubt the presence of (Ba,Sr)SO_4_ solid solutions. The ν_1_(SO_4_) band was observed at 990 ± 2, 994 ± 2 and 1000 ± 2 cm^−1^, which correspond to a stoichiometric composition of (Ba_0.88_Sr_0.12_)SO_4_ (green regions), (Ba_0.5_Sr_0.5_)SO_4_ (blue regions) and Ba_0.05_Sr_0.95_SO_4_ (red regions), respectively, with an uncertainty in the mole fraction of Ba of ± 0.1. (Ba_0.88_Sr_0.12_)SO_4_ was detected only in chamber 1 (crystal 1 at the very early stages, and crystal 3 post clogging at the pillars) over a limited region, while all other crystals exhibited a bimodal solid solution composition distribution with alternating compositions (Ba_0.5_Sr_0.5_)SO_4_ and Ba_0.05_Sr_0.95_SO_4_.

**Figure 4:**
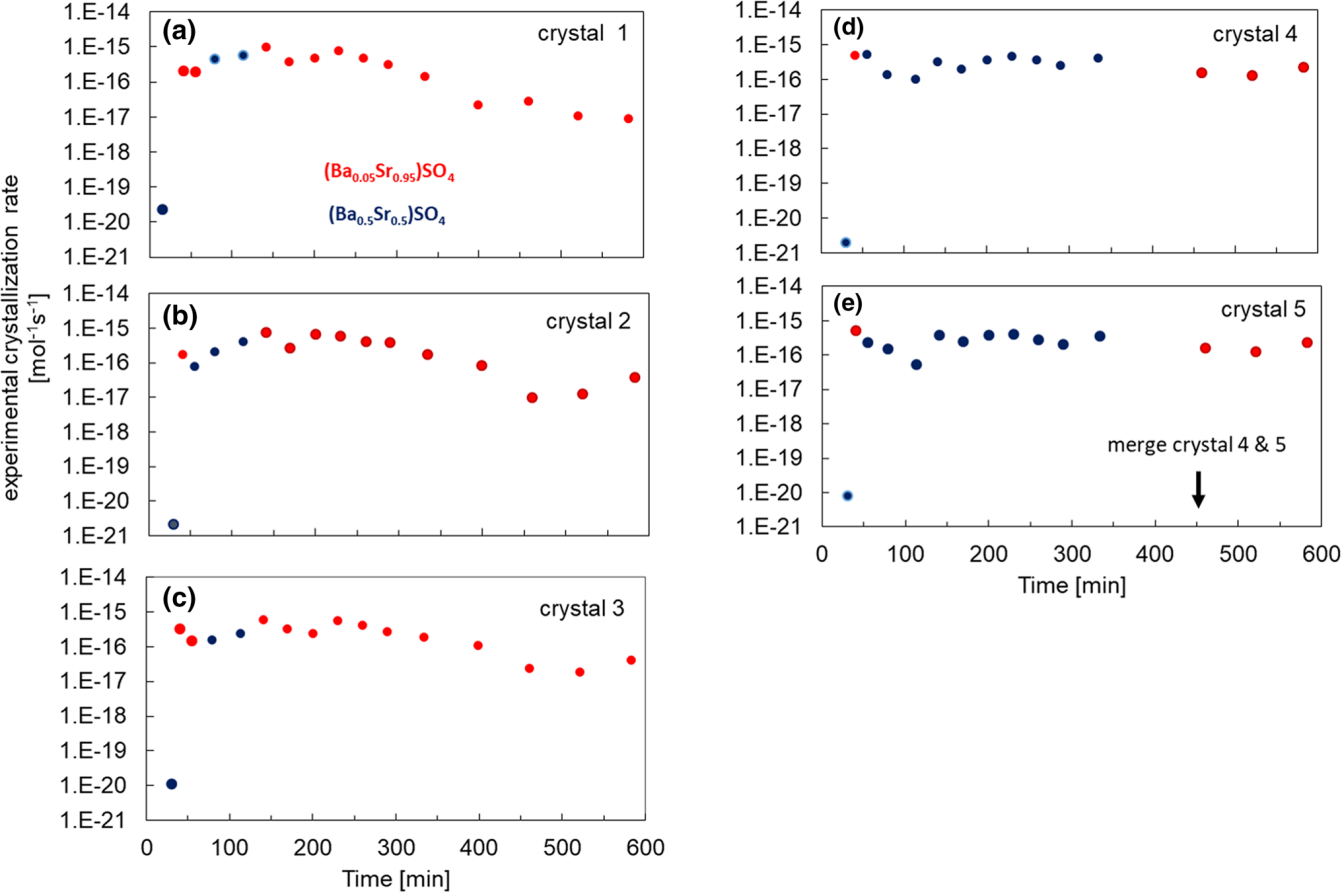
(**a**–**e**) experimentally derived crystallization rates of crystals 1–5 in reaction chamber 4.

The crystal growth rates of the individual crystals were calculated from the time lapse images as discussed in the Methods Section (see also supplement S5); the results for chamber 4 are presented in Fig. 4. The growth rates are more or less constant between 30 and 300 min. Afterwards, as the pathways for solute exchange get obstructed due to the growing crystals, the growth rates for crystals 1, 2 and 3 decrease, while no significant change is observed for crystals 4 and 5. Prior to clogging, the average crystallization rates of Ba_0.5_Sr_0.5_SO_4_ and Ba_0.05_Sr_0.95_SO_4_ of each crystals in chamber 4 were evaluated at 2.9 ± 0.8 × 10^−16^ and 4.5 ± 0.8 × 10^−16^ mol s^−1^, respectively. The total amount of minerals that precipitated per unit time is 1.8 ± 0.1 × 10^−15^ mol s^−1^.

### Evaluation of the flow field and determination of the dominant mass transport mechanisms with in the growth chamber at steady state

**Figure 5:**
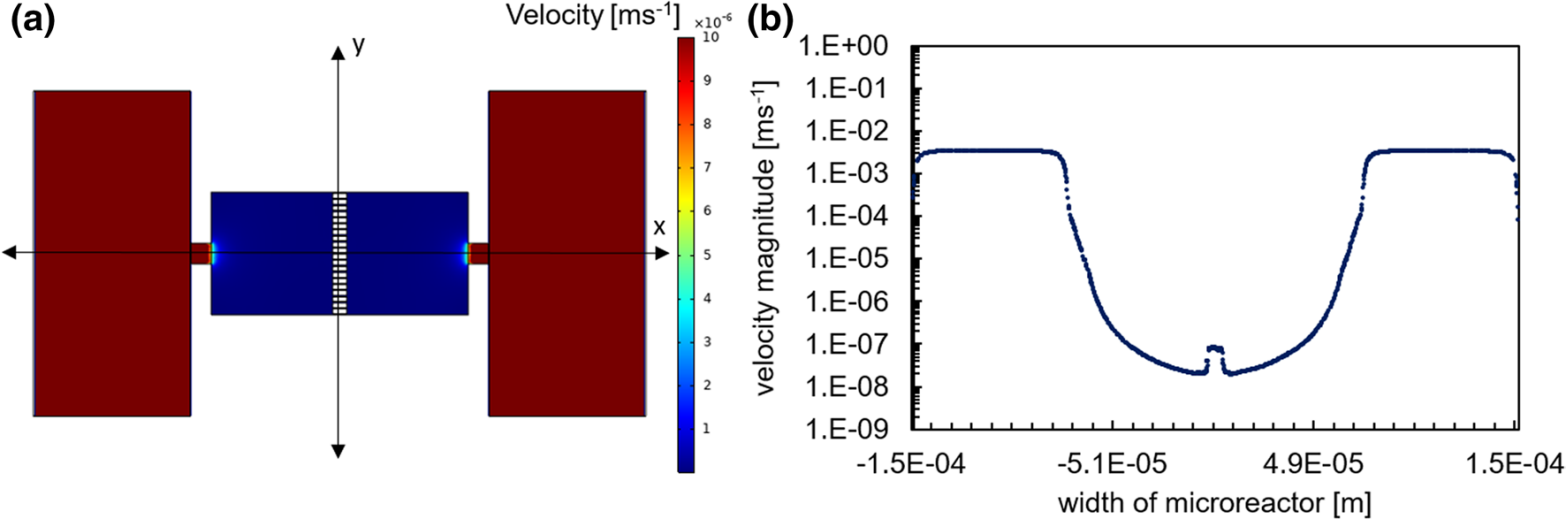
(**a**) Distribution map of the velocity magnitude at steady state, and (**b**) profile of the velocity magnitude along line y = 0 with the center of the growth chamber as origin.

The injection of solutions at constant rate in the supply channels generates a velocity field at steady state as shown in Fig. 5a. The velocity magnitude along the line y = 0 is depicted in Fig. 5b. It shows that the velocity is highest at ~ 1.2 × 10^−3^ ms^−1^ in the solution supply channels and decreases hyperbolically to ~ 5 × 10^−7^ ms^−1^ in the center of the reactor. The Peclet number (*P*_e_, defined as the ratio of advective to diffusive transport rates) within the growth chamber was evaluated at 0.01, after considering a characteristic length of 100 μm (i.e. a length over which the velocity is in the same order of magnitude). It can thus be inferred that diffusion dominates the transport of solutes within the growth chamber.

### Determination of the local concentrations and saturation indices at the initial stage of the experiment

2D pore scale lattice Boltzmann simulations were conducted using the exact photographic images. This resulted in a two-dimension lattice grid composed of 245 × 100 grid points and a resolution of 0.6 μm. The time step of the simulation was Δt = 4.28 × 10^−5^ s. The machine learning accelerated geochemical speciation^23^ was used at every time step and every grid point, thus providing the necessary input for the calculation of the saturation indices.

**Figure 6:**
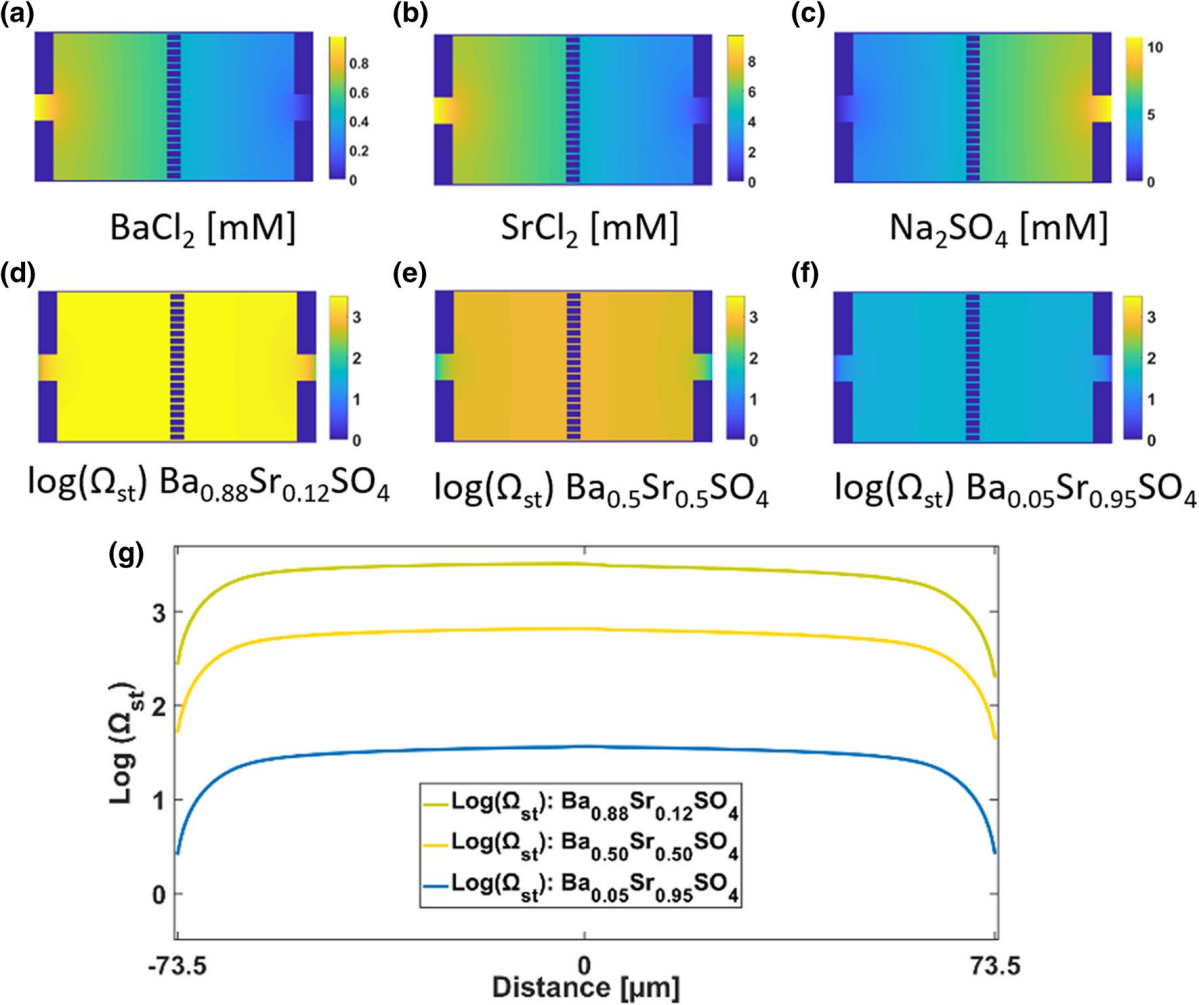
Map of (**a**) BaCl_2_, (**b**) SrCl_2_ and (**c**) Na_2_SO_4_ concentrations distributions in the growth chamber and associated stoichiometric supersaturation ratio with respect to (**d**) Ba_0.88_Sr_0.12_SO_4_, (**e**) Ba_0.5_Sr_0.5_SO_4_ and (**f**) Ba_0.05_Sr_0.95_SO_4_ with their respective plots of stoichiometric saturation ratio profiles along y = 0 in (**g**) for a better visualization.

The distribution of the concentrations of BaCl_2_, SrCl_2_ and Na_2_SO_4_ at steady state (without precipitation reactions) is shown in Fig. 6. A concentration gradient builds up 30 s after the start of the injection, with the highest concentrations at the respective supply channel, which decreases to zero in the opposite channel. This concentration gradient reaches a steady state in the absence of chemical reactions. The distribution of Ba^2+^, Sr^2+^ and SO_4_^2+^ at the entrance of the growth chambers follows concentric circles which shade off further away from the inlet. Indeed, the solute concentrations do not vary significantly along the y axis for − 50 µm < x < 50 µm. The simulated SI (log Ω) with respect to the observed precipitating phases are shown in Fig. 6d–f, respectively. The saturation ratio for the Ba rich phase (i.e., X = 0.88 and X = 0.5) is slightly higher in the left compartment of the growth chamber, while the highest saturation ratio with respect to the strontium rich phase occurs in the central part of the growth chamber (Fig. 6g).

Previous work^22^ has shown that the boundary conditions at the entrance of the first five growth chambers vary less than 0.1%. Therefore, we assume the same variation of boundary conditions at the entrance of the first 5 chambers in the present set-up.

### Continuum scale modelling of the transport induced precipitation

For a better understanding of the mechanisms that drive the zoning phenomena, we conducted two comparative modelling studies simulating the precipitation of Ba_0.5_Sr_0.5_SO_4_ and Ba_0.05_Sr_0.95_SO_4_ with two different methods: (1) precipitation assuming instantaneous equilibration (case study 1) and (2) precipitation with kinetic (case study 2) in a simplified 1D geometry, using a continuum-scale reactive transport model solved with OGS-GEMS.

**Figure 7:**
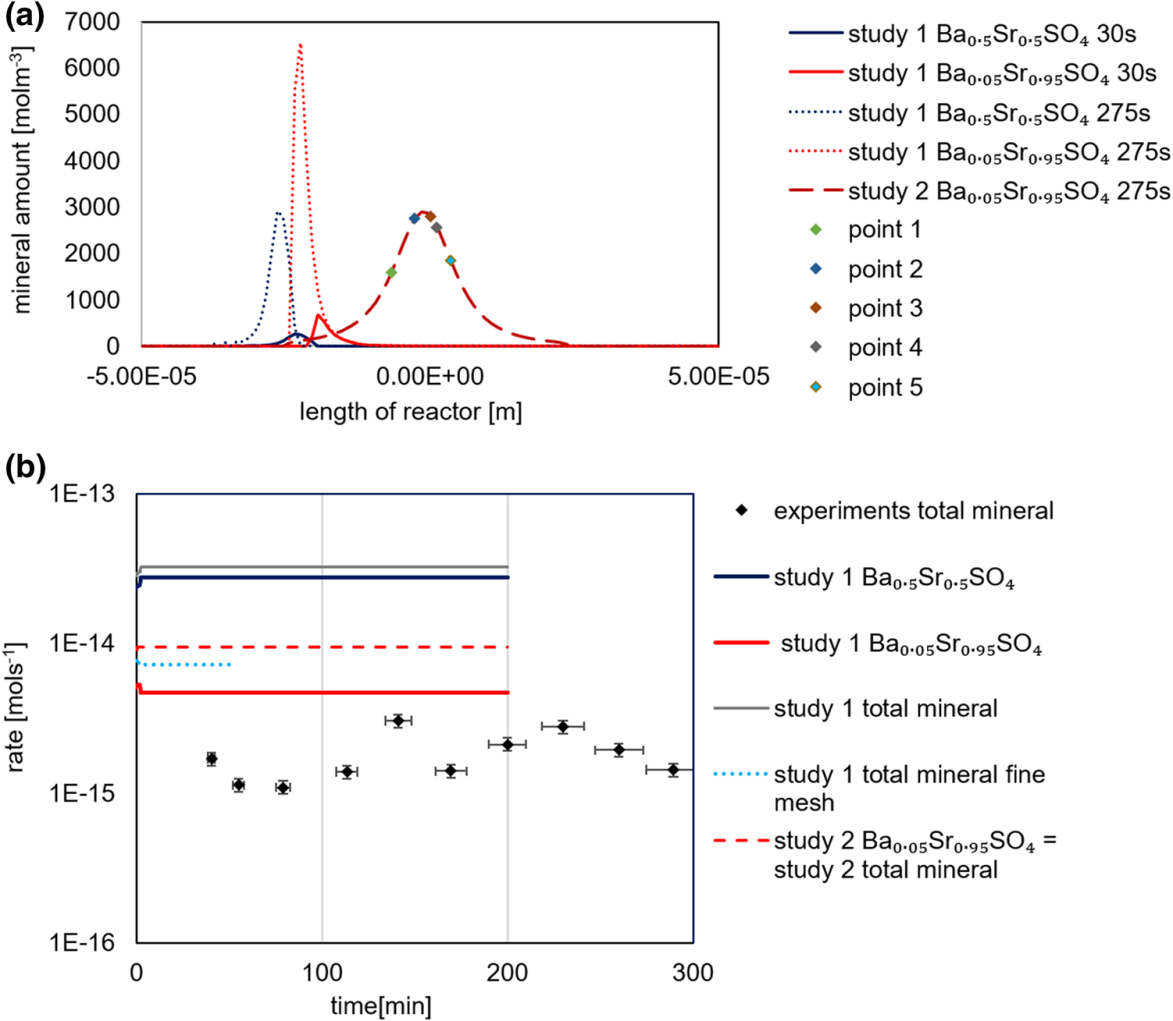
(**a**) Mineral distribution profiles of the two stoichiometric compositions (Ba_0.05_Sr_0.95_)SO_4_ and (Ba_0.5_Sr_0.5_)SO_4_ across a simulated 1D reactor for case study 1 at 30 and 275 s and case study 2 at 275 s using a discretization of 1 µm. Points 1–5 refer to the sampling points used to calculate nucleation and crystallization kinetics in Fig. 9. (**b**) Comparison of simulated precipitation rates using OGS-GEM with instantaneous precipitation (case study 1) and with kinetic constraints (case study 2) using mesh discretizations of 5 µm and 1 µm against experimental results (total amount of minerals that precipitated per unit time).

Figure 7a shows the predicted mineral precipitation across the 1D column for case study 1 and 2, respectively. In case study 1, both experimentally observed Ba_0.5_Sr_0.5_SO_4_ and Ba_0.05_Sr_0.95_SO_4_ precipitated. The precipitation starts in the left compartment, where the initial concentrations of Ba^2+^ and the stoichiometric supersaturation ratio with respect to Ba_0.5_Sr_0.5_SO_4_ (Fig. 6) are higher_._ In study 1, continuous re-equilibration between the solid phases and aqueous solution is allowed, which explains the shift in the two maxima between the time 30 and 275 s in Fig. 7a. The re-equilibration is due to the dissolution of Ba_0.5_Sr_0.5_SO_4_ in favour of the precipitation of Ba_0.05_Sr_0.95_SO_4_. The sum of the associated rate of precipitation is one order of magnitude higher (3.3 × 10^−14^ mol s^−1^) than the observed experimental rates (1.8 ± 0.1 × 10^−15^ mol s^−1^) (Fig. 7b). Mesh discretization is known to impact the simulated amounts of minerals that precipitate per unit time^40^. Therefore, we refined the mesh discretization to 1 µm and simulated the evolving system for 50 min. Our sensitivity analysis of study 1 (Fig. 7b) showed that the simulated amount of minerals that precipitated per unit time for case study 1 was lower (~ 7.3 × 10^−15^ mol s^−1^) in this case, but still higher than the experimental rates. Two major outcomes are the results of this study. First, for the conditions of the experiment, diffusion coupled with thermodynamic equilibration results in large over prediction of the precipitation rates, verifying that the observed phenomena are not transport (diffusion) limited, and that the interplay of kinetics play a significant role. Second, such a modelling provides not only quantitative, but also qualitative very different results compared to the experimental observations. In contrast to experimental observations, there are no precipitates forming preferentially at the right compartment only.

When the kinetics of precipitation of the two stoichiometric compositions are considered (case study 2), only Ba_0.05_Sr_0.95_SO_4_ precipitated in the simulation (Fig. 7a). The precipitation of Ba_0.5_Sr_0.5_SO_4_ is kinetically hindered because of the lower kinetic constant of precipitation (~ 1 order of magnitude). A higher simulated precipitation rate of BaS_0.05_r_0.95_SO_4_ (9 ± 0.1 × 10^−15^ mol s^−1^) in case study 2 compared to study 1 is observed and explained by the higher saturation ratios that can build up in the system since no other phase precipitates. In case study 2, the process is rate limited and therefore the mesh discretization between 1 and 5 µm has little impact on the amounts of minerals that precipitate per unit time and is therefore not shown here. The applied kinetic model for the precipitation of solid solutions cannot describe our experimental observations but provide better qualitative description of the location of precipitates (i.e. in the middle of the growth chamber).

## Discussion

### Composition of the nucleating phase

**Figure 8:**
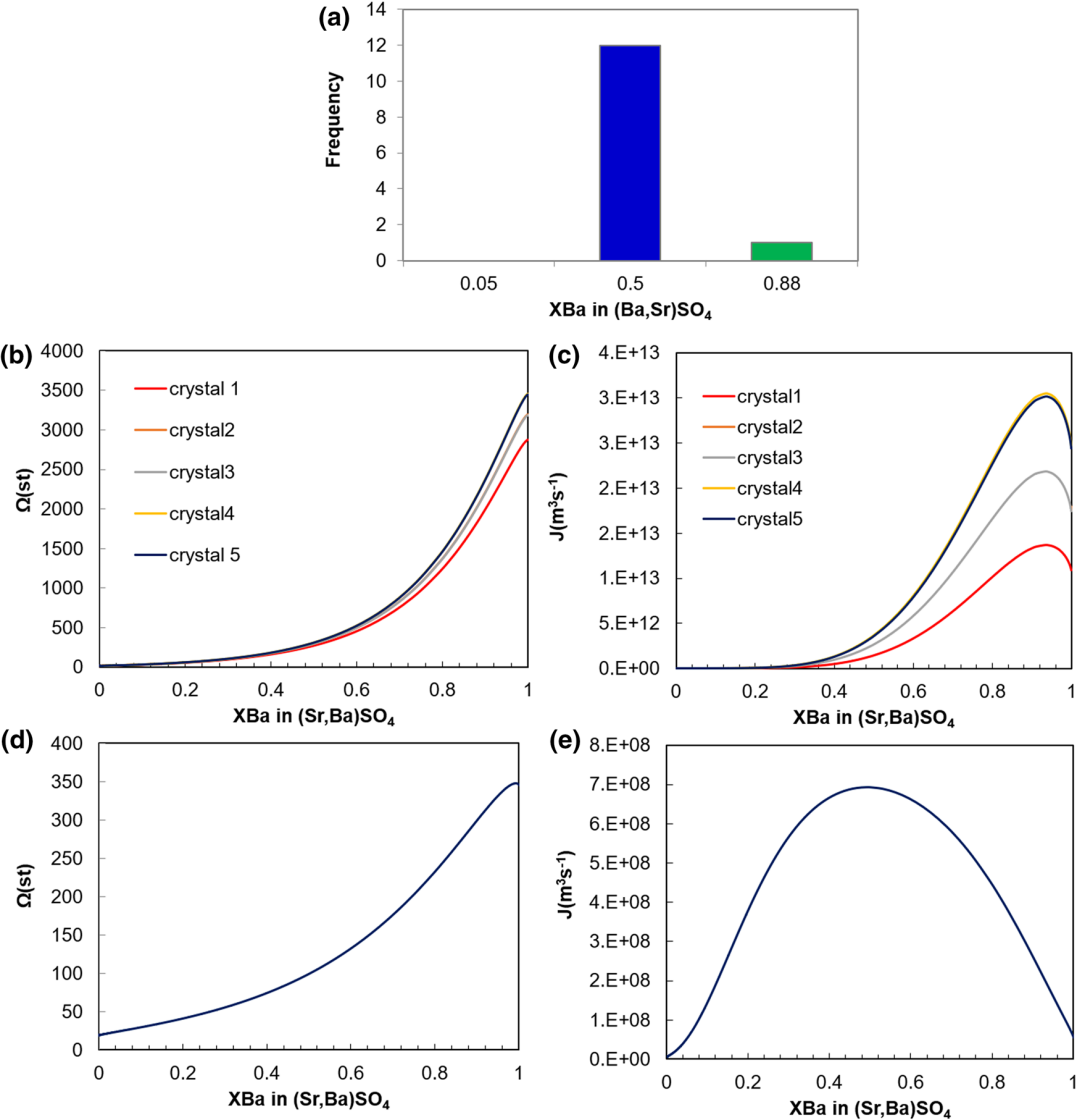
(**a**) Histograms of the solid solution compositions sampled in the first 5 growth chambers, (**b**) stoichiometric supersaturations computed for the aqueous solution compositions at the locations where crystals 1–5 in chamber 4 start to nucleate (aSr^2+^/aBa^2+^ ~ 10) and (**c**) corresponding nucleation rates, (**d**) stoichiometric supersaturation function and e) associated nucleation rate for a hypothetical aqueous solution composition with aSr^2+^/aBa^2+^ ~ 100.

The stoichiometric composition of the nucleating phase (i.e. of the core of crystallites) in the 5 monitored microfluidic chambers was (Ba_0.5_Sr_0.5_)SO_4_ except for crystal 1 in chamber 1 (see Fig. 4), where a composition of (Ba_0.88_Sr_0.12_)SO_4_ was also observed. Figure 8a reports the frequency of occurrence of the different compositions observed in the crystals during Raman mapping.

The concentrations and activities of Ba^2+^, Sr^2+^ and SO_4_^2−^ at the location where the crystallites nucleated were extracted from our lattice Boltzmann simulations (Fig. 6) to calculate the stoichiometric supersaturation functions and associated nucleation rates on the surface of PDMS. Note that the concentrations used for this analysis correspond to the initial stage of the experiment were precipitation is not yet initiated and is used here as a first approximation of the local concentrations after the crystal growth. The results for chamber 4 are shown in Fig. 8b–e. The supersaturation functions for crystal 2 and 3 practically superpose each other since the aqueous compositions are almost identical (both crystals are located on same x-coordinate value). A similar effect applies to crystals 4 and 5. The supersaturation functions show that the solution at the nucleation locations is supersaturated with the entire range of possible solid solution compositions (Ω > 1 for *X*_Ba_ = 0 to 1) and therefore any composition could potentially precipitate. The maxima of these functions lie at *X*_Ba_ = 0.99. This is the solid composition with respect to which supersaturation is highest and it corresponds to true thermodynamic equilibrium with the specific aqueous composition. The computations using the saturation state of the solution using the δ function gave similar results^28^ (see supplement S6 for more details). The nucleation rates were computed using classical nucleation theory and the outcome for chamber 4 is presented in Fig. 8c with the maxima (*X*_Ba_ = 0.93) indicating the solid solution composition with the highest probability of nucleation. At thermodynamic equilibrium, a (Ba_0.99_Sr_0.01_)SO_4_ enriched solid solution should precipitate while the kinetic CNT approach suggests nucleation of a Ba-poorer solid with composition (Ba_0.93_Sr_0.07_)SO_4_. However, only once among the 13 analyzed spots a similar composition, (Ba_0.88_Sr_0.12_)SO_4_ with *X*_Ba_ ± 0.1) was experimentally observed.

This apparent inconsistency may be explained by taking into account that the nucleation process could induce changes in the local composition of the aqueous solution in direct contact with the crystals. Shortly after nucleation, the formation of an almost pure BaSO_4_ cluster might lead to a quick local decrease in aqueous Ba concentration. This would considerably increase the ratio of the aqueous Sr (injected in excess) to Ba. In a hypothetical case in the vicinity of the aforementioned event and by considering that the Sr/Ba ratio in the aqueous solution could increase to 100, the maximum of the stoichiometric saturation function would shift only slightly (Fig. 8d), but the probability of nucleating (Ba_0.5_Sr_0.5_)SO_4_ would dominate over other stoichiometric compositions (Fig. 8e). After the nucleation on the surface of PDMS, further nucleation on the surface of existing sulphates is much faster indicated by the shorter induction time (see Table 1) because of the decrease in surface tension. The formation of (Ba_0.93_Sr_0.07_)SO_4_ is possible and most likely limited in time (takes place at the very early stages only) and space and is therefore not detected at the resolution of our measurement techniques. Moreover, there is ample evidence that demonstrate that the composition of the nuclei is frequently not determined by the maximum supersaturation values alone^15^.

**Table 1:** Calculated effective surface tension σ (*X*_Ba_) for different substrates and nucleating phases and calculated induction times^41^.

Substrate	Nucleating phase	σ (*X*_Ba_) effective (Jm^−2^)	Calculated induction time (s)
PDMS	Ba_0.88_Sr_0.12_SO_4_	0.077	4.6
PDMS	Ba_0.5_Sr_0.5_SO_4_	0.067	35.6
Ba_0.88_Sr_0.5_SO_4_	Ba_0.5_Sr_0.5_SO_4_	0.017	4 × 10^−6^

Beside by the stoichiometric saturation function and nucleation kinetics, the composition of the precipitated phase can also be determined by the partition coefficient concept^8^. The partition coefficient concept implies that at very fast precipitation rates, all cations in contact with the growing mineral will be trapped by the layer of newly formed precipitate^42^ such that the composition of the growing phase has the same composition as the surrounding solution, which in our experiment would be (Sr_0.9_Ba_0.1_)SO_4_. The current observation does not follow this trend and it can therefore be inferred that the growth rates are relatively low.

Our results indicate that the composition of the nucleating phase is most likely controlled by nucleation kinetics and can be addressed using Classical Nucleation Theory. Our experimental results are in agreement with the experimental work of Prieto et al.^13^, where (Ba,Sr)SO_4_ zoning is typically bimodal with an expansion of the number of compositions ((Ba_0.88_Sr_0.12_)SO_4_ and Ba_0.5_Sr_0.5_SO_4_)) at higher supersaturation all while keeping the bimodal behaviour.

### Towards a mechanistic understanding of the zoning process

The presented continuum scale modelling scenarios cannot reproduce our experimental observations. Case study 1 with instantaneous precipitation overestimated the mineral precipitation rates, suggesting that the precipitation of oscillatory zoned crystals of (Ba,Sr)SO_4_ is controlled by kinetics. In case study 2, which includes the crystal growth rates of minerals, the rate of precipitation was still higher than the experimental rates and the formation of Ba_0.5_Sr_0.5_SO_4_ was kinetically suppressed. It can thus be inferred that the precipitation of Ba_0.5_Sr_0.5_SO_4_ is controlled by processes that are kinetically slower and which are not included in the presented model, e.g., nucleation processes.

**Figure 9:**
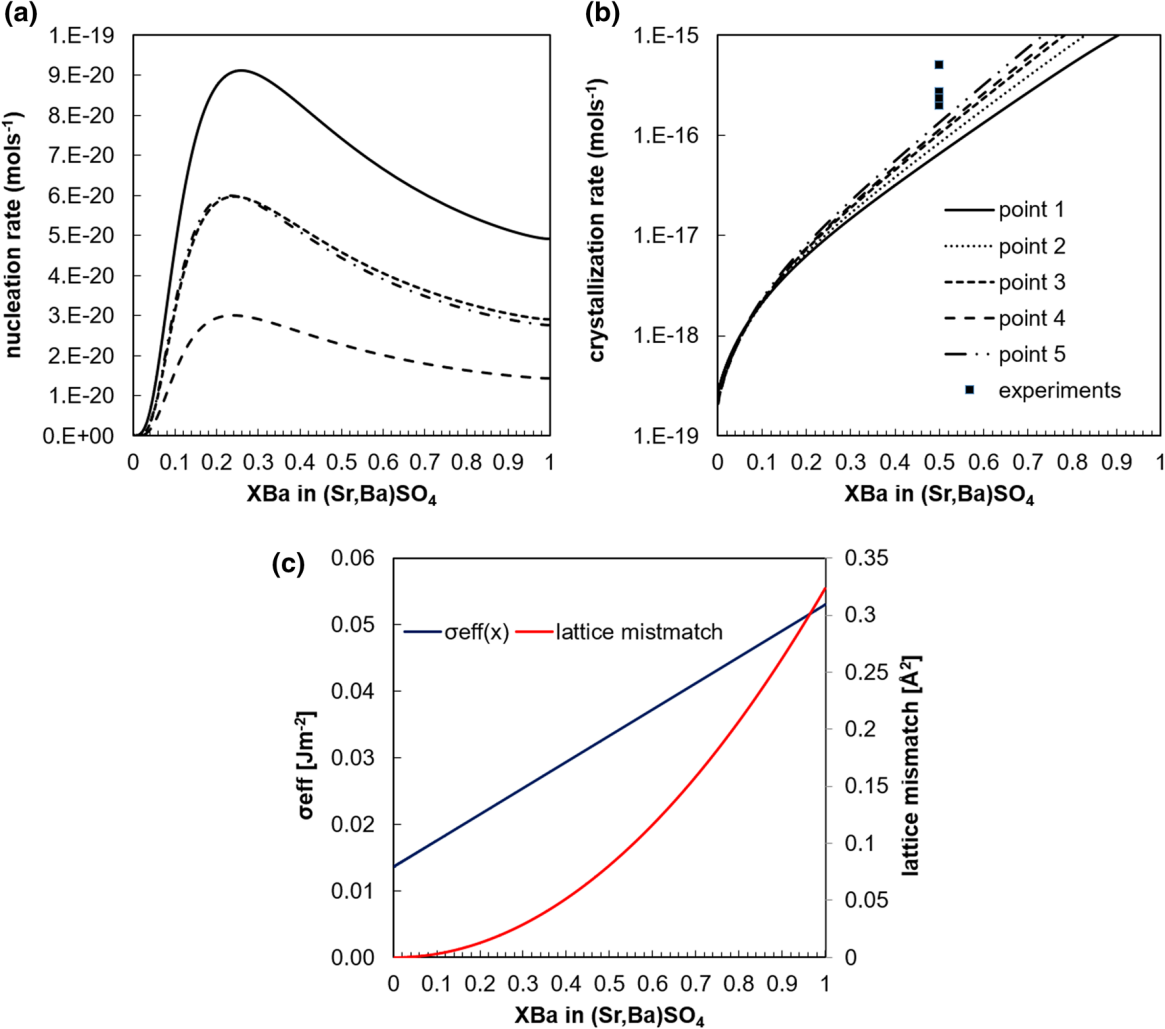
(**a**) Nucleation rates, (**b**) associated crystallization rates, and (**c**) calculated effective surface tension (primary vertical axis) and lattice mismatch (secondary vertical axis) as function of the solid solution series.

To verify this hypothesis, we sampled the simulated aqueous solution composition at different locations (points 1–5 in Fig. 7a in case study 2) to compute the rate of nucleation and crystallization of (Ba,Sr)SO_4_ solid solutions on the surface of Ba_0.05_Sr_0.95_SO_4_ (Fig. 9). The sampled points typically reflect the solution composition after the precipitation of Ba_0.05_Sr_0.95_SO_4_. The solution chemistry with aSr^2+^/aBa^2+^ ~ 2, indicates that further nucleation of Ba_0.05_Sr_0.95_SO_4_ is kinetically hindered but that other compositions with an increased Ba content can nucleate (shown by the maxima of the curves for points 1–5 in Fig. 9a). The precipitation of barium enriched phases is kinetically favoured via nucleation mechanisms. After the precipitation of Ba_0.05_Sr_0.95_SO_4_, the nucleation of barium enriched solid solutions becomes competitive enough to allow their precipitation. In fact, the switch to stoichiometric phases other than Ba_0.05_Sr_0.95_SO_4_ could be explained by the fact that the nucleation rate of the other stoichiometric phases becomes higher than the rate of crystal growth of Ba_0.05_Sr_0.95_SO_4_. This successive nucleation and crystal growth operating at different rates (mol s^−1^) can also explain the experimentally observed oscillating crystallization rates in Fig. 7b.

The solution chemistry after the precipitation of Ba_0.05_Sr_0.95_SO_4_ favours crystallization of an almost pure barite (Fig. 8b), however, crystallization of solid solutions with compositions with X_Ba_ > 0.6 on the surface of Ba_0.95_Sr_0.05_SO_4_ is unlikely and was observed only once and after clogging at the pillars (crystal 3 in chamber 1), where the concentration of aqueous barium can build up in the system. Other factors related to the crystallography of the solid solutions could be decisive for the determination of the composition of the solid solutions that can precipitate. For instance, lattice mismatch (equation S7.1 in supplement S7) that characterizes the difference between the lattice parameters of the growing solid solution and its substrate. Due to significant differences in lattice parameters between the growing phase and the substrate (Fig. 9c), only specific phases can precipitate. Such phenomena are common for the epitaxial growth of sulphate solid solutions^9^. Similarly, the surface tension and therefore the energetic barrier associated with the growth of solid solutions with X_Ba_ > 0.6 on the surface of Ba_0.95_Sr_0.05_SO_4_ is relatively large, and is therefore unlikely to occur.

The 1D continuum approach is limited to describe the processes occurring at the mineral-solution interfaces, nevertheless, it provides strong evidence that oscillatory zoned crystals of (Ba,Sr)SO_4_ are not solely the result of a diffusion induced precipitation process but rather depends on crystallization kinetics. The inclusion of crystal growth kinetics alone cannot capture the composition of the precipitating phases but other processes such as nucleation could explain the observed slower precipitation rates. Nucleation mechanisms, although not considered in many reactive transport codes, can be important nanoscale mechanisms influencing macroscale phenomena in subsurface environments^43^.

Another aspect which is expected to play significant role is the spatial heterogeneity especially when the precipitates start to grow. The shape and location of the crystals will alter the diffusion transport fluxes and pathways, while the total reactive surface area (solid/aqueous solution interface) will be continuously evolving, certainly passing through diffusion limited evolution stages before the eventual porosity clogging. In the future, to address the complexity and interplay of mass transport and reaction mechanisms at the pore-level we will focus in the development of a digital twin based on pore-scale lattice Boltzmann framework with machine learning enhanced geochemical speciation. This will enable a systematic assessment of the interplay of mass transport and crystallization kinetics of solid solutions (i.e. the sum of nucleation rate and crystal growth rate) to resolve the physics at the crystal/solution interfaces by taking into account the true crystal heterogeneity as has been done previously for pure celestine^22,23^ and barite^38^.

## Summary

We developed a lab on a chip experiment that enabled the systematic in-situ assessment of oscillatory zoned crystals of (Ba,Sr)SO_4_ in a confined volume. Our investigations showed that the composition of the nucleating phase can be determined using classical nucleation theory extended to solid solutions. Our numerical and modelling studies were conducted at different levels, each providing significant information for the interpretation of the experiments. The continuum approach modelling, suggested that oscillatory zoning is not the result of the limited diffusion of solutes at least at the first stages. The switch between phases with different stoichiometry is likely caused by competition between nucleation and crystal growth of the two stoichiometric phases. Beside kinetics, other factors such as lattice mismatch or surface tension can also play an important role for the determination of the precipitating phases. In future work, we will integrate crystallization kinetics of solid solutions in our lattice Boltzmann model to create a digital twin of the system to resolve the geochemical processes at the mineral-solution interfaces to overcome the shortcoming of the applied approaches.

## Supplementary Information

Supplementary Video 1.

Supplementary Information 1.
