# Supplementary material for: A lab-on-a-chip approach integrating in-situ characterization and reactive transport modelling diagnostics to unravel (Ba,Sr)SO4 oscillatory zoning
Source: Sci Rep. 2021 Dec 8;11:23678. doi: 10.1038/s41598-021-02840-9 (PMC8654837; doi:10.1038/s41598-021-02840-9)
Supplement: Supplementary file 2 — Supplementary Information 1. [file 41598_2021_2840_MOESM2_ESM.docx]

# Supplements

## Supplement S1: Evaluation of kinetic constants for the (Ba,Sr)SO_4_ solid solution series

Experimental data on the precipitation kinetics of (Ba,Sr)SO_4_ solid solutions is scarce and not reported in literature. Here, we evaluate the kinetic rate of solid solution formation based on the experimental dataset of Weber et al. (2018), where the authors combined a set of analytical techniques, i.e. scanning electron microscopy with energy dispersive X-ray fluorescence spectrometry (STEM-EDS), atom probe tomography (APT), time of –flight secondary ion mass spectrometry (TOF-SIMS), and hydrothermal atomic force microscopy (HAFM) to determine the (Ba,Sr)SO_4_ solid solution composition and associated growth rates. The experimental data was collected at a temperature of 381.15 K and under an inflow of N_2_. A summary of the experimental data is provided in Table S1.1. In our data evaluation, we excluded the TEM experimental results because the quantification of the solid solution composition is unreliable while the quantification using atom probe is more realistic.

The precipitation rate of barite is known to follow a second order reaction (Bosbach et al. 2002). For solid solutions, the kinetic constant is not a single value but a function of the solid solution composition. Assuming that the precipitation rate *r*_s_ [mol s^-1^] for a given composition of a (Sr,Ba)SO_4_ solid solution also follows a second order reaction, the kinetic constant, *k*_s_, of a given stoichiometry can be calculated using the relationship:

$R=\frac{r}{S_{s}}=k_{s}({1-\Omega_{s})}^{2}$ (S1.1)

where, $R$ is the normalized crystal growth rate reported in Table S1a, *S*_s_, the reactive surface area [m^2^], and $\Omega_{s}$ is the saturation state, as the associated $\Omega_{s}$to the measured solid solution composition.

The saturation state of the aqueous solutions used by Weber et al. (2018) with respect to the solid solution that had precipitated was computed from the composition of the aqueous solutions reported in Table S1.1 using two methodologies: (i) the stoichiometric saturation function and the (Ω_st_), and (ii) the δ functions of Astilleros et al. (2003).

The supersaturation, (Ω_st_), as function of the solid solution composition at a temperature of 381.15K (and in inert atmosphere) was calculated for each solution mixture listed in Table S1.1 using equation S1.2 (Prieto et al. (2009)):

$Ω_{st}(X_{Ba})=\frac{{(a_{{Ba}^{2+}})}^{X_{Ba}}{(a_{{Sr}^{2+}})}^{X_{Sr}}(a_{{{SO}_{4}}^{2-}})}{\left( K_{BaSO_{4}}\gamma_{BaSO_{4}}X_{Ba} \right)^{X_{Ba}}.\left( K_{SrSO_{4}}\gamma_{SrSO_{4}}X_{Sr} \right)^{X_{Sr}}}$ (S1.2)

where $a_{{Ba}^{2+}}$, $a_{{Sr}^{2+}}$ and $a_{{{SO}_{4}}^{2-}}$ represent the activities in the free solution and were computed using GEMS considering the extended Debye-Huckel ionic strength activity model; $K_{BaSO_{4}}$ and $K_{SrSO_{4}}$, the solubility products of the end-members BaSO_4_ and SrSO_4_ (with K_sp_(BaSO_4_)=10^-9.58^ and K_sp_(SrSO_4_)=10^-7.32^ for a temperature of at 381.15 K taken from Hummel et al. (2002)); and $X_{BaSO_{4}}$ and $X_{SrSO_{4}}$, the molar fractions of BaSO_4_ and SrSO_4_ in the solid. $\gamma_{BaSO_{4}}$ and $\gamma_{SrSO_{4}}$ are the activity coefficients of the end members in the solid solution and were calculated using the Thompson-Waldbaum-Model assuming a regular mixing model with a Margules interaction parameter, *w*, of 4950 J mol^-1^ (Vinograd et al. 2018):

$RTln\gamma_{BaSO_{4}}\approx w{{\times X}_{SrSO_{4}}}^{2}$ (S1.3)

$RTln\gamma_{SrSO_{4}}\approx w{{\times X}_{BaSO_{4}}}^{2}$ (S1.4)

The δ functions of Astilleros et al. (2003) are given as:

$\partial_{BaSO_{4}}\left( X_{Ba} \right)=\frac{{[Ba}^{2+}][{SO}_{4}^{2-}]}{K_{BaSO_{4}}\gamma_{BaSO_{4}}X_{Ba}}$ for $X_{Ba}\geq X^{max}$, (S1.5)

and

$\partial_{SrSO_{4}}\left( X_{Ba} \right)=\frac{{[Sr}^{2+}][{SO}_{4}^{2-}]}{K_{SrSO_{4}}\gamma_{SrSO_{4}}X_{Sr}}$ for $X_{Ba}\leq X^{max}$ (S1.6)

The maximum of the stoichiometric saturation ratio and the meeting points of the delta function represent the thermodynamically most stable phase for a given aqueous solution composition and are the same.

Table S1.1: Summary of the experimental data of Weber et al. (2018) and calculated saturation state of the solutions.

| Name used in Weber et al. (2018) | [Ba^2+^]_aq_  mol L^-1^ | [Sr^2+^]_aq_  mol L^-1^ | [SO_4_^2-^]_aq_  mol L^-1^ | Growth rate normalized to surface area  R  µmol m^-2^ s^-1^ | composition of solid solution | **saturation state Ω_s_**  Cal. as Ω_st_  in this work | **Saturationsstate**  **Ω_s_** Cal as δ  in this work |
| --- | --- | --- | --- | --- | --- | --- | --- |
| SrBa0 HAFM 1 | 3.68×10^-5^ | 0 | 3.63×10^-5^ | 3.0×10^-4^ | BaSO_4_ | 3.16 | 3.98 |
| SrBa0 HAFM 2 |  |  |  | 2.1×10^-4^ |  | 3.16 | 3.162 |
| SrBa0.5 HAFM 1 | 3.68×10^-5^ | 2.02×10^-5^ | 3.63×10^-5^ | 2.0×10^-4^ | (Ba_0.97_Sr_0.03_)SO_4_ | 3.94 | 0.095 |
| SrBa0.5 HAFM 2 |  |  |  | 2.0×10^-4^ |  | 3.94 | 0.095 |
| SrBa0.5 APT 1 |  |  |  | 3.8×10^-4^ |  | 3.94 | 0.095 |
| SrBa0.5 APT 2 |  |  |  | 3.4×10^-4^ |  | 3.94 | 0.095 |
| SrBa1 HAFM 1 | 3.68×10^-5^ | 3.64×10^-5^ | 3.63×10^-5^ | 1.6×10^-4^ | (Ba_0.96_Sr_0.04_)SO_4_ | 3.40 | 0.11 |
| SrBa1 HAFM 2 |  |  |  | 6.3×10^-5^ |  | 3.40 | 0.11 |
| SrBa1 APT 1 |  |  |  | 2.1×10^-4^ |  | 3.40 | 0.11 |
| SrBa1 APT 2 |  |  |  | 2.0×10^-4^ |  | 3.40 | 0.11 |
| SrBa5 APT 1 | 4.08×10^-5^ | 2.22×10^-4^ | 3.93×10^-5^ | 3.1×10^-5^ | (Ba_0.88_Sr_0.12_)SO_4_ | 2.90 | 0.28 |
| SrBa5 APT 2 |  |  |  | 9.0×10^-5^ | (Ba_0.94_Sr_0.06_)SO_4_ | 3.4 | 0.43 |
| SrBa16 APT | 4.7×10^-5^ | 7.3×10^-3^ | 4.3×10^-5^ | 1.5×10^-4^ | (Ba_0.6_Sr_0.4_)SO_4_ | 1.62 | 0.4 |
| SrBa22 APT | 4.7×10^-5^ | 1×10^-3^ | 4.2×10^-5^ | 1.5×10^-4^ | (Ba_0.6_Sr_0.4_)SO_4_ | 1.62 | 0.4 |
| SrBa44 APT | 5.1×10^-5^ | 2.4×10^-4^ | 4.2×10^-5^ | 1.5×10^-4^ | (Ba_0.6_Sr_0.4_)SO_4_ | 1.46 | 0.7 |
| SrBa68 APT | 5.1×10^-5^ | 4.8×10^3^ | 3.9×10^-5^ | 1.5×10^-4^ | (Ba_0.6_Sr_0.4_)SO_4_ | 1.14 | 0.8 |

The associated Ω_s_ for each solid solution that precipitated was calculated and tabulated in Table S1.1. Using the growth rates R and Ω_s_ (from the two different method of calculation in Table S1) in equation S1.1, the kinetic constants for precipitation of the measured solid solution compositions were calculated for a temperature of 381.15 K and plotted in Figure S1.2 (left). Using the Arrhenius equation (S1.5), the kinetic constants at 298.15K were calculated and plotted as function of the solid solution composition in Figure S1.2 (right).

$ln\frac{k_{381.15K}}{k_{298.15K}}=-\frac{Ea}{R}\left( \frac{1}{T_{381.15K}}-\frac{1}{T_{298.15K}} \right)$ (S1.5)

The activation energies, E_a_, of precipitation of pure BaSO_4_ and SrSO_4_ have been reported as 35±8 kJ mol^-1^ (Bosbach et al. 2002) and 34 kJ mol^-1^(Marty et al. 2012), respectively. Consequently, in our evaluation of the rate constants, the activation energy was assumed to be constant (35 kJ mol^-1^) for all solid solution compositions. N.B. we noticed that the δ calculated from the solution composition from Weber et al. (2018) is < 1 suggesting that the solution was in fact undersaturated and might therefore be not the best way to evaluate the saturation ratio for the experiments of Weber et al. 2018 since precipitation was observed. We however processed and plotted the results only for the sake of completeness.
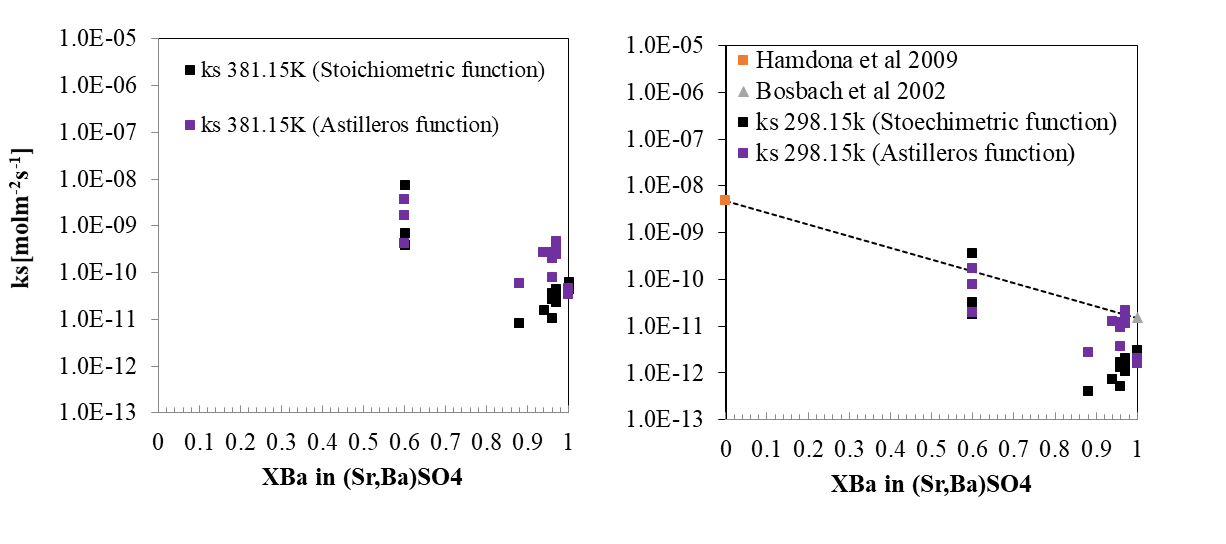


Figure S1.2: Kinetic constants as function of solid solution composition at 381.15 K (left) and 298.15 K (right). The kinetic constant for pure celestine was re-evaluated following a second order reaction using the experimental data of Hamdona et al. 2009.

Based on the measurements of Weber et al. (2018), we assume that the kinetic constant (*k*_s_) for precipitation is a linear function of the solid solution composition with *k*_s_ equal to 4.8×10^-9^ and 1. 5×10^-11^ mol s^-1^ for X_Ba_=0 and X_Ba_=1, respectively.

**References**

J. M. Astilleros, C. Pina, L.Fernandez-Diaz and A. Putnis, "Spersaturation functions in binary solid solution-aqeous solution systems," Geochimica Cosmochimica Acta, vol. 67, no. 9, pp. 1601-1608, 2003.

D. Bosbach, "Linking molecular-scale barite precipitation mechanisms with macroscopic crystal growth rates," in In: Water-Rock Interactions, Ore deposits and Environmental Geochemistry: A tribute to David A Crerar, The Geochemical Society, R.Hellmann and S.A Wood, 2002, pp. 97-110.

S. Hamdona and S. Hamza, "Influence of polyphosphonates on the precipitation of strontium sulfate (celestite) from aqueous solutions," J. Taibah Univ. Sci., vol. 2, pp. 36-43, 2009.

W. Hummel, U. Berner, E. Curti, F. Pearson and T. Thoenen, "Nagra/PSI Chemical Thermodynamic Data Base 01/01," Radiochimica Acta, vol. 90, pp. 805-813, 2002.

N. Marty, F. Claret, A. Lassin, J. Tremosa, P. Blanc, B. Made, E. Giffaut, B. Cochepin and C. Tournassat, "A database of dissolution and precipitation rates for clay-rocks minerals.," Appl. Geochem. , vol. 55, pp. 108-118., 2015.

M. Prieto, "Thermodynamics of solid solution-aqueous solution systems," Reviews in Mineralogy and Geochemistry, vol. 70, pp. 47-85, 1 2009.

V. Vinograd, D. Kulik, F. Brandt, M. Klinkenberg, J. Weber, B. Winkler and D. Bosbach, " Thermodynamics of the solid solution—aqueous solution system (Ba,Sr,Ra)SO_4_ + H_2_O: I. The effect of strontium content on radium uptake by barite," Applied Geochemistry, vol. 89, pp. 54-74, 2018.

J. Weber, J. N. Bracco, J. D. Poplawsky, A. V. Ievlev, K. L. More, M. Lorenz, A. L. Bertagni, S. A. Jindra, V. Starchenko, S. R. Higgins and A. G. Stack, "Unraveling the effects of strontium incorporation on barite growth - In situ and ex situ observations using multiscale chemical imaging," Crystal Growth and Design, vol. 18, pp. 5521-5533, 2018.

## Supplement S2: Modelling of the fluid dynamics with the Finite Elements Method (FEM) using COMSOL Multiphysics

Numerical simulations were used to assess the local streamlines and velocity magnitudes in the microfluidic chamber. This knowledge allows us to relate the different precipitation mechanisms to the transport mechanisms in the micromodel. The transport equations were solved using the computational fluid dynamics software COMSOL Multiphysics 5.3a (COMSOL AB, Stockholm, Sweden). The velocity field in the reactor was calculated using the Navier-Stokes equation for laminar flow of an isothermal, incompressible and Newtonian fluid:

$\rho u\cdot\nabla u=-\nabla p+\mu\nabla^{2}u ; \nabla\cdot u=0$ (S3.1)

where u is the velocity vector, ρ and µ are the density and viscosity of the fluid (water) set to 1000 kg m^-3^ and 8.90 × 10^−4^ Pa s, respectively. A no-slip condition was applied to the glass and PDMS walls of the reactor and a laminar inflow with a flow rate of 500 nL min^-1^ was specified for each of the two supply channel inlets. A reference pressure of 0 Pa was applied to both outlets.

The transport of solute equation is given as:

$0=-u\cdot\nabla C_{i}+\nabla\left( D_{i}\nabla C_{i} \right)$ (S3.2)

where $C_{i}$ (i=1, 2, 3…m) (mol m^-3^) denotes the molar concentration of the i^th^ species of an m multi-species system; u is the velocity (m s^-1^) of the fluid flow, *D*_i_ is the diffusion-dispersion coefficient of component i set to 1.23×10^-9^ m^2^ s^-1^ for Na_2_SO_4_ (average for Na^+^ (1.33×10^-9^ m^2^s^-1^) and SO_4_^2-^ (1.07×10^-9^ m^2^ s^-1^)), 1.34×10^-9^ m^2^s^-1^ for SrCl_2_ (average for Sr^2+^ (7.94×10^-9^ m^2^ s^-1^) and Cl^-^ (2.03×10^-9^ m^2^ s^-1^)) and 1.39×10^-9^ m^2^ s^-1^ for BaCl_2_ (average for Ba^2+^ (8.47×10^-9^ m^2^ s^-1^) and Cl^-^ (2.03×10^-9^ m^2^ s^-1^)). This step enables the determination of the time when steady state is reached and of the initial concentration gradients in the system. Our modelling in this step considers neither the aqueous speciation of the solutes nor chemical reactions.

## Supplement S3: 1D reactive transport model simulations using OpenGeoSys GEM

**Model setup**


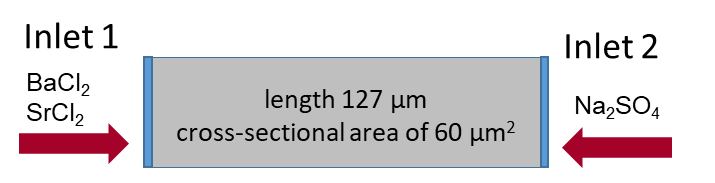


Figure S4.1: Schematics of the 1D model

The 1D model (shown in Figure S4.1) was resolved using OpenGeoSys-GEM (Shao et al., 2009; Kosakowski and Watanabe, 2014). The fluid flow and mass transport equations (equations S4.1 and S4.2) are solved by OpenGeoSys based on a standard finite element formulation, whereas the chemical processes are solved by the GEMS3K kernel code of GEM-Selektor V3 (Kulik et al., 2013).

The reactive transport equations according to Bear and Bachmat (1990) are given as:

$\frac{dC_{i}}{dt}=\nabla\left( D_{i}\nabla C_{i} \right)+Q_{i}$ (S3.1)

$\frac{dC_{i}}{dt}=ᴦ_{i}(C_{i}\ldots C_{m})$ (S3.2)

where $C_{i}$ (i=1, 2, 3…m) (mol m^-3^) denotes the molar concentration of the i^th^ species of an m multi-species system; *D*_i_ is the diffusion-dispersion coefficient of component i (assumed to have the same value for all components in our model); Q_i_ is the source/sink term and ᴦ_i_(C_i_…C_m_) is the source/sink term of species i due to chemical reactions with other species.

The experiment was modelled in a 1-D geometry discretized with a grid size of 5-6 or 1-3 µm. The properties of the setup and the boundary and initial conditions used for the numerical simulations are summarised in Table S3.1. The characteristic time scales for transport were evaluated and are summarised in Table S3.2. We verified that the chosen time steps respect the von Neumann criteria to avoid any physically unrealistic solutions, such as negative concentrations and numerical oscillations.

The solubility products of the two solid solutions with the stoichiometric compositions (Ba_0.5_Sr_0.5_)SO_4_ and (Ba_0.05_Sr_0.95_)SO_4_ were calculated as 4.12×10^-9^ and 1.43×10^-7^ mol^2^ L^-2^, respectively, using the following expression:

$K_{st}= \left( K_{XBa}X_{XBa} \right)^{X_{XBa}}.\left( K_{Xsr}X_{XSr} \right)^{X_{Xsr}}.exp(\frac{\Delta G_{E}}{RT})$ (S3.2)

where $\Delta G_{E}$ is the excess Gibbs energy of mixing for the non-ideal solid solution:

$\Delta G_{E}=RT(X_{Ba}ln\gamma_{Ba}+X_{Sr}ln\gamma_{Sr})$ (S3.3)

$K_{st}= \left( K_{XBa}{\gamma_{Ba}X}_{XBa} \right)^{X_{XBa}}.\left( K_{Xsr}\gamma_{Sr}X_{XSr} \right)^{X_{Xsr}}$ (S3.4)

$K_{BaSO_{4}}$ and $K_{SrSO_{4}}$, the solubility products of the end-members BaSO_4_ and SrSO_4_ for a temperature of at 298,15 K taken from Hummel et al. (2002)); and $X_{BaSO_{4}}$ and $X_{SrSO_{4}}$, the molar fractions of BaSO_4_ and SrSO_4_ in the solid. $\gamma_{BaSO_{4}}$ and $\gamma_{SrSO_{4}}$ are the activity coefficients of the end members in the solid solution

The reaction rates,$r_{(XBa)}$ [mol s^-1^], of dissolution and precipitation processes were calculated following Palandri and Kharaka (2004). The rates of dissolution and precipitation were calculated as

$r_{(XBa)}={S_{s}k}_{XBa}({1-\Omega_{st(XBa)})}^{2}$ (S3.5)

where $S_{s}$ [m^2^] is the reactive surface area of the precipitating phase. In our simulations, reactive surface areas are calculated as follows

$S_{s}=V\times a$ (S3.6)

where *V* [m^3^] is the volume of the mineral, *a* [m^2^ m_mineral_^-3^] is a mineral’s specific surface area (i.e., surface area per volume of the mineral phase) set to 50000 m^2^ m_mineral_^-3^ , which is the average of the experimentally measured reactive surface area for the first 200 minutes.

Table S6.1: Transport related properties of the 1D setup.

| Characteristics |  |
| --- | --- |
| Length [m] | 127×10^-6^ |
| Initial porosity $\Phi$_0_ [-] | 0.99 |
| Pore diffusion coefficient D_e_ [m^2^s^-1^] | 9.9×10^-10^ |
| Cross-sectional area [m^2^] | 60×10^-12^ |
| Inlet 1 composition & concentrations | 1 mM BaCl_2_ 9.9 mM SrCl_2_ |
| Inlet 2 composition & concentration | 10.9 mM Na_2_SO_4_ |
| Initial pH fixed by partial pressure of CO_2_ | 5.6 |
| Modelling time duration (minutes) | 200 |
| Reactive surface area a [m^2^] |  |

Table S6.2: Characteristic time scales for transport and chemical processes for the numerical model.

| Quantity | value |
| --- | --- |
| size discretization Δx [m] | 6×10^-6^; 23*{5×10^-6^}; 6×10^-6^ |
| Von Neumann criterion [s], $time step<\frac{{\Delta x}^{2}}{{2D}_{e}}$ | <0.0125 |
| Damköhler number,${S_{s}k}_{XBa}\times\frac{{\Delta x}^{2}}{D_{e}}$  5 | <<1 |

## Supplement S4: 2D Pore-scale simulations

Figure S4.1: Simulation domain of the 2D pore-scale calculations


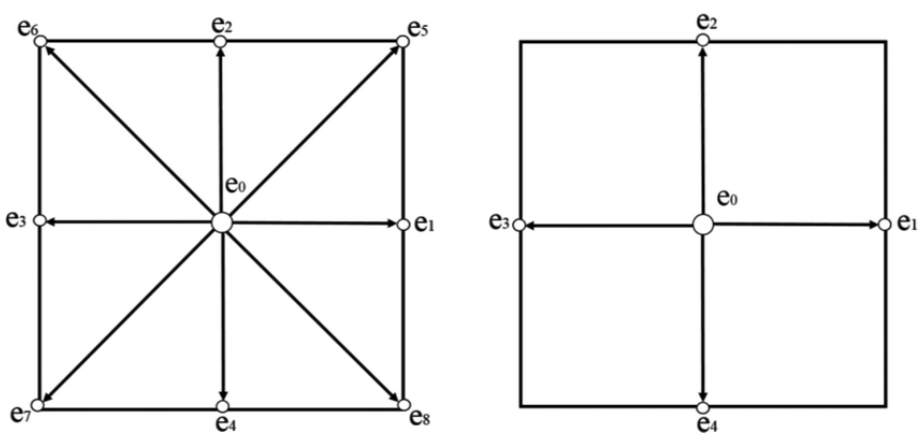


Figure S4.2: The D2Q5 velocity lattice

In the 2D pore-scale model, the solutes transport was performed using the lattice Boltzmann method. The simulation domain (shown in Figure S4.1) was imported directly from the photographic image of the reaction chamber, where each pixel in the image is represented by a D2Q5 lattice point (shown in Figure S4.2). The injected solutions are transported via diffusion by solving the discretized lattice Boltzmann transport equation with BGK approximation, given by[1];

$$f_{i}\left( x+e_{i}\Delta t , t+\Delta t \right)=f_{i}\left( x, t \right)+ \frac{f_{i}^{eq}\left( x, t \right) - f_{i}\left( x, t \right)}{\tau} \Delta t$$

where $f_{i}\left( x, t \right)$ is the discretized distribution function representing solutes moving along velocity vector $“i”$ from location $“x”$ at time step $“t”$ with velocity $“e_{i}”$ to a neighboring point of location $“x+e_{i}\Delta t”$ at the next time step $“t+\Delta t”$. $\tau$ is the relaxation time, set equal to unity. $f_{i}^{eq}\left( x, t \right)$ is the discretized equilibrium distribution function, given by;

$$f_{i}^{eq}\left( x, t \right)=w_{i}C$$

where $w_{i}$ are the lattice constants. For the D2Q5, the weights for the five velocity vectors (e0, e1, e2, e3, e4) are [2/6 , 1/6 , 1/6 , 1/6 , 1/6]. $C$ is the solute concentrations, calculated from;

$$C(x,t)=\sum_{i=0}^{4} f_{i}(x,t)$$

The above equations are solved for each of the injected solutions, namely BaCl_2_, SrCl_2_, and Na_2_SO_4_. The inlet and outlet of each injected solution were treated as constant concentration boundary conditions. The solid/fluid boundaries were treated with a half-way bounce-back method. The model was initialized with zero concentrations everywhere except at the inlet, where the concentrations are set according to the experimental conditions. The concentration gradient between the inlet and the outlet drives each solution to diffuse slowly across the chamber, until steady state is achieved around 30 seconds.

After the transport step using LBM, the full speciation of the injected solutions at each lattice point is calculated using a pre-trained geochemical speciation surrogate model [2 & 3]. From the known speciation, the saturation index is calculated using the stoichiometric saturation function;

$$\Omega(X_{Ba})=\frac{{\{{Ba}^{+2}\}}^{X_{Ba}}{. \{{Sr}^{+2}\}}^{X_{Sr}} . \{{SO}_{4}^{-2}\}}{\left( K_{BaSO_{4}}\gamma_{BaSO_{4}}X_{Ba} \right)^{X_{Ba}}.\left( K_{SrSO_{4}}\gamma_{SrSO_{4}}X_{Sr} \right)^{X_{Sr}}}$$

where $\{{Ba}^{+2}\}$, $\{{Sr}^{+2}\}$, and $\{{SO}_{4}^{-2}\}$ are the activities of ${Ba}^{+2}$, ${Sr}^{+2}$, and ${SO}_{4}^{-2}$ ions, respectively. $K_{{sp}_{BaSO_{4}}}$and $K_{{sp}_{SrSO_{4}}}$are the solubility product constant of barite ($BaSO_{4}$) and celestine ($SrSO_{4}$) minerals, set equal to ${10}^{-9.97}$ and ${10}^{-6.63}$, respectively. $X_{Ba}$ and $X_{Sr}$ are the mole fraction of the pure phases $BaSO_{4}$ and $SrSO_{4}$, respectively. $\gamma_{BaSO_{4}}$ and $\gamma_{SrSO_{4}}$ are the activity coefficients of the pure phases $BaSO_{4}$ and $SrSO_{4}$, respectively, calculated by;

$$\gamma_{BaSO_{4}}= X_{Ba}*exp( \left( 2A_{0}-A_{1} \right)*{X_{Sr}}^{2}+2\left( A_{1}-A_{0} \right)*{X_{Sr}}^{3})$$

$$\gamma_{SrSO_{4}}= X_{Sr}*exp( \left( 2A_{1}-A_{0} \right)*{X_{Ba}}^{2}+2\left( A_{0}-A_{1} \right)*{X_{Ba}}^{3})$$

where $A_{0}$ and $A_{1}$ are the dimensionless Thompson-Waldbaum parameters given by;

$$A_{1}=\frac{w_{bar/cel}}{RT}$$

$$A_{0}=\frac{w_{cel/bar}}{RT}$$

where $w_{bar/cel}$ and $w_{bar/cel}$ are the Margules parameters set both equal to 4950 J/mol, $R$ is the universal gas constant in J/mol.k, and T is the temperature in Kelvin.

[1] Krüger, Timm, et al. "The lattice Boltzmann method." *Springer International Publishing* 10.978-3 (2017): 4-15.

[2] De Lucia, Marco, et al. "Integrating surrogate models into subsurface simulation framework allows computation of complex reactive transport scenarios." *Energy Procedia* 125 (2017): 580-587.

[3] Prasianakis, Nikolaos I., et al. "Neural network based process coupling and parameter upscaling in reactive transport simulations." *Geochimica et Cosmochimica Acta* 291 (2020): 126-143.

## Supplement S5: Evaluation of crystal growth rates in chamber 4


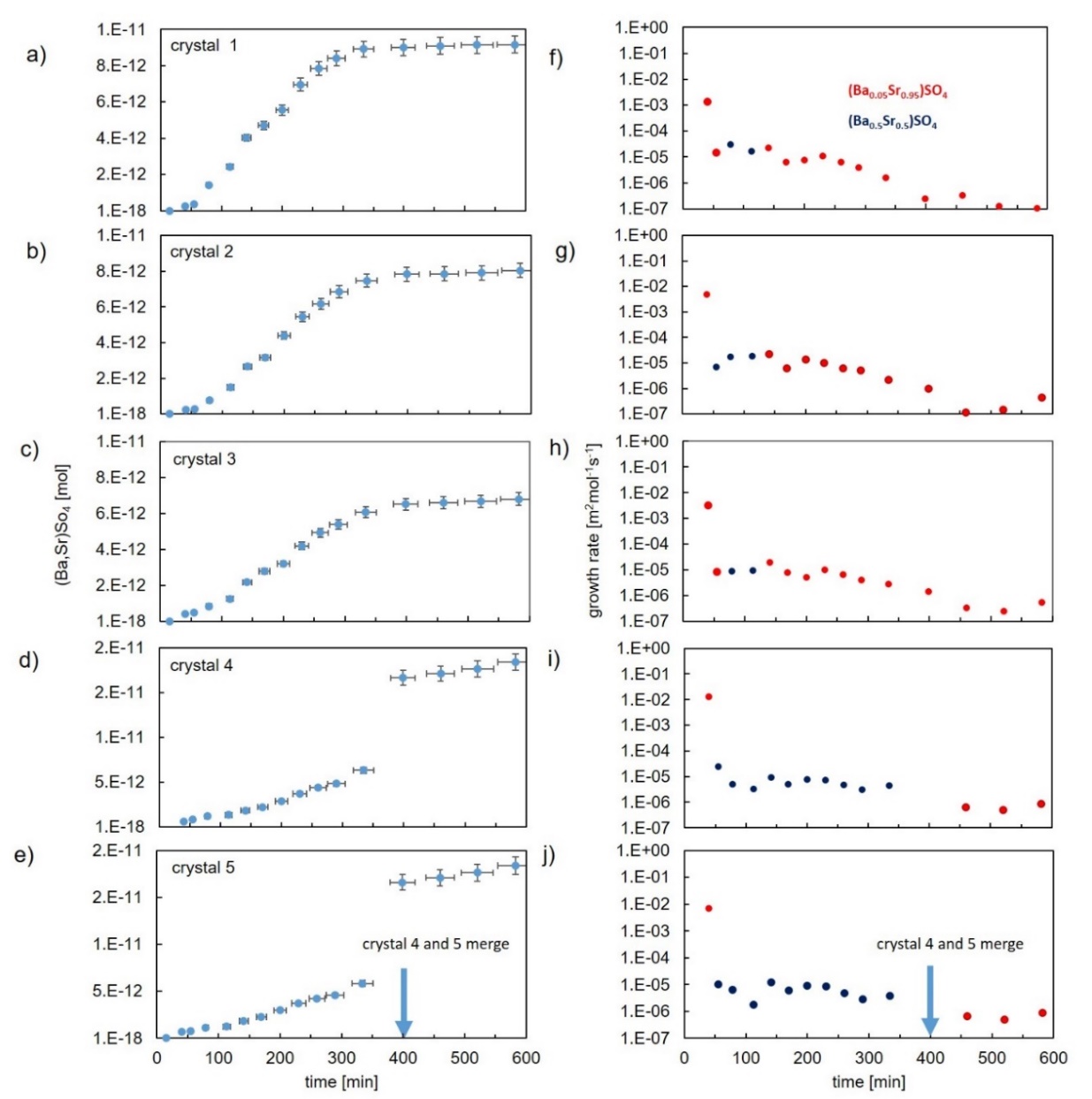


Figure S5.1: a), b), c), d) and e) are the amounts of precipitates and associated crystals growth rates f) g) h) i) and j) of the crystals in chamber 4 labelled 1 to 5, respectively. After 400 minutes, crystal 4 and 5 merges and the rate of growth of the merged crystal is plotted.

The growth rates of the crystals labelled 1 to 5 (Figure 3 in main document) of chamber 4 were evaluated and plotted against time in Figure S5.1. Crystal 1 grows faster than the other crystals, which is indicated by the amount of moles that precipitated per unit time. For the first 50 minutes the growth rates (normalized to the surface area) lie between 10^-2^ and 10^-3^ molm^-2^s^-1^ and then decrease to a rather constant rate of 5×10^-6^ molm^-2^s^-1^ to 400 minutes whereby clogging at the pillars is observed and the growth rates decrease. The rates measured between 50 and 400 minutes for (Ba_0.5_Sr_0.5_)SO_4_ are in the same order of magnitude as those calculated for a supersaturation function of 3000 (i.e. 1.8×10^-12^ molm^-2^s^-1^×(1-3000)^2^ = 1.6×10^-5^). The supersaturation function w.r.t to Ba_0.05_Sr_0.95_SO_4_ is likely to vary between 20 and 50 and the estimated rate is 1.7×10^-6^ and 1.2×10^-5^ molm^-2^s^-1^ which is in agreement with our experimental observations.

## Supplement S6: Computation of nucleation rates using the δ Astilleros function


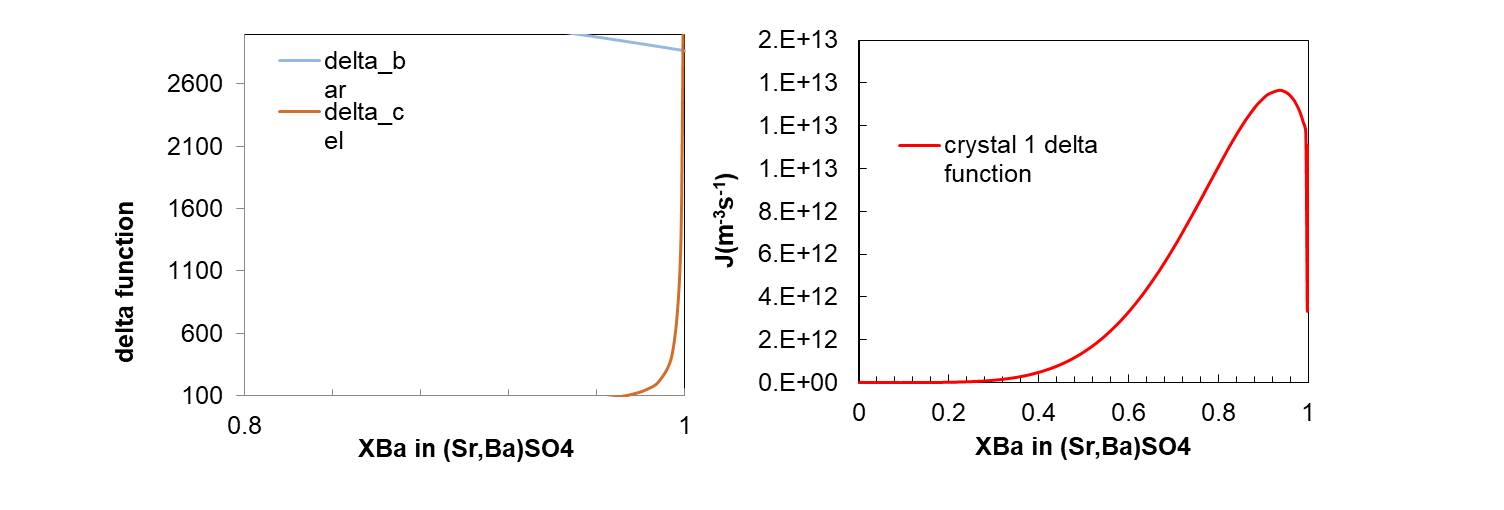


Figure S6.1: a) Computation of the saturation state using the delta function and b) associated nucleation rate for crystal 1 in chamber 4.

The saturation state of the solution can also be calculated using the δ function, whereby the thermodynamic most stabled phase is in agreement with the saturation state function at X_Ba_ = 0.99. The nucleation rate in CNT computed using the δ function indicates a higher probability of nucleation for X_Ba_ = 0.94.

## Supplement S7: Computation of lattice mismatch

For a given solid solution of Ba_1-x_Sr_x_SO_4_, growing on a surface_,_ the lattice mismatch is given as (Klinkenberg et al. 2018):

$${R^{2}=(a_{x}-a}_{0})^{2}+{(b_{x}-b}_{0})^{2}{+(c_{x}-c}_{0})^{2}$$

where a, b, c are the lattice parameters and the indices x and 0 refer to the precipitating phase and the substrate respectively. We assume a linear change of the lattice parameters with the composition

The lattice parameters used in our calculations are tabulated in Table S7.1.

Table S7.1: Lattice parameters of pure barite and celestine according to Vinograd et al. (2018).

V. Vinograd, D. Kulik, F. Brandt, M. Klinkenberg, J. Weber, B. Winkler and D. Bosbach, "Thermodynamics of the solid solution—aqueous solution system (Ba,Sr,Ra)SO_4_ + H_2_O: I. The effect of strontium content on radium uptake by barite," Applied Geochemistry, vol. 89, pp. 54-74, 2018.

## Supplement S8: Raman spectra of the (Ba,Sr)SO4 solid solution series

| **EDX measurement of x in**  **Ba_x_Sr_1-x_SO_4_** | **Raman Band**  ν_1_(SO_4_) band maxima |
| --- | --- |
| 0.06±0.01 | 999 |
| 0.18±0.01 | 998 |
| 0.5±0.04 | 994 |
| 0.73±0.05 | 992 |

(Ba,Sr)SO_4_ solid solutions synthesized by flux synthesis from Brandt et al. 2018 and 2020, previously characterized by EDX (see Fig S8.1) were analyzed by Raman spectroscopy. The Raman spectra are given in figure S8.2 and the positions (cm^−1^) of the ν_1_(SO_4_) band maxima as function of the mole fraction of both end members.


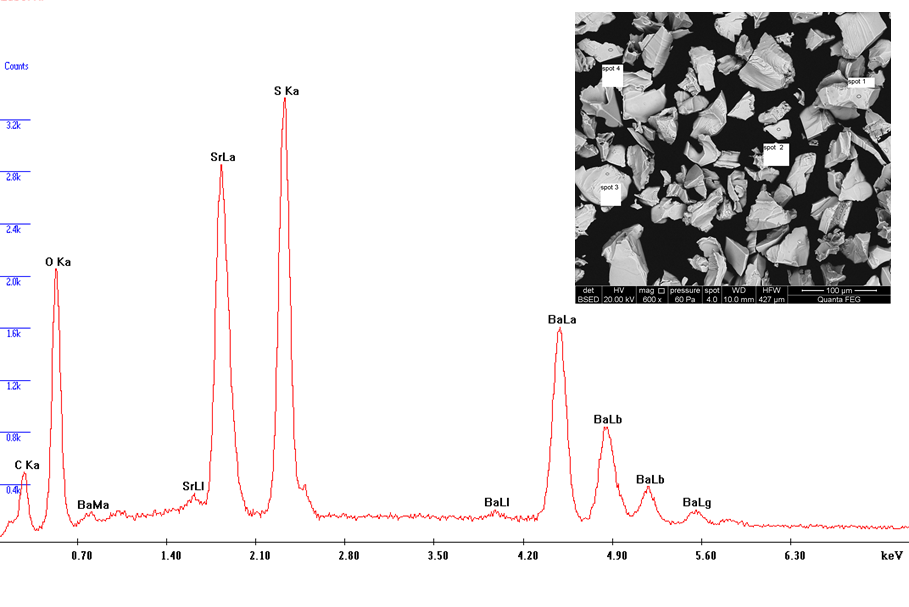


Fig S8.1: EDX measurement of single crystals of Ba_0.5_Sr_0.5_SO_4_.


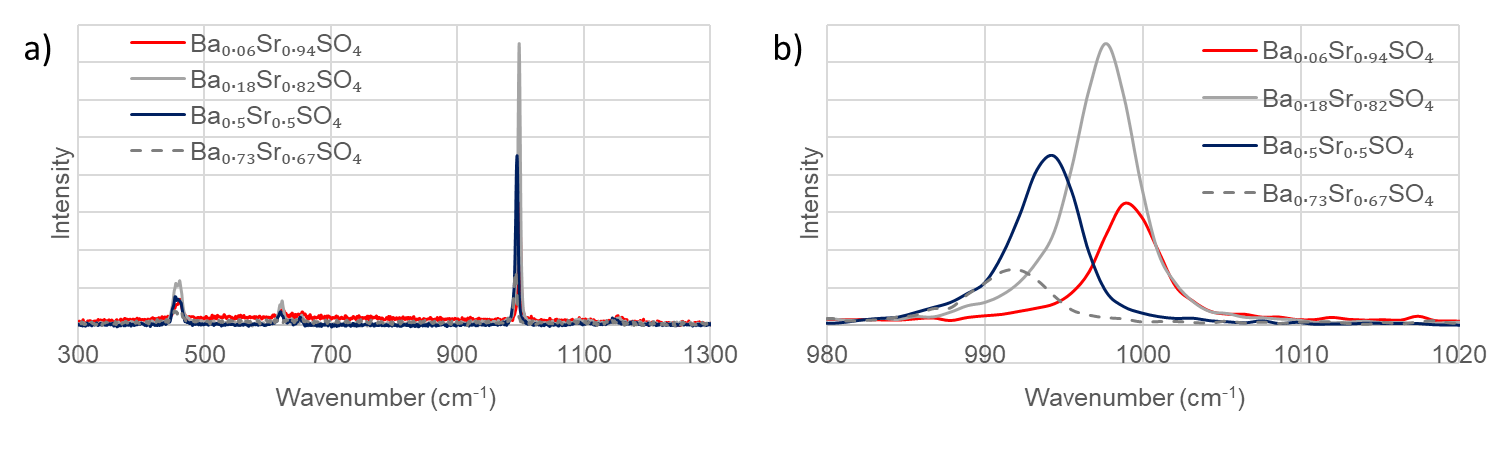


Fig. S8.2: a) Raman spectra of samples synthesized by flux synthesis and previous analyzed by SEM-EDX with b) a zoom on the ν_1_(SO_4_) band.

Brandt F, Klinkenberg M, Poonoosamy J, Weber J, Bosbach D. The Effect of Ionic Strength and Sr_aq_ upon the Uptake of Ra during the Recrystallization of Barite. Minerals. 2018; 8(11):502. <https://doi.org/10.3390/min8110502>

Brandt, F.; Klinkenberg, M.; Poonoosamy, J.; Bosbach, D. Recrystallization and Uptake of ^226^Ra into Ba-Rich (Ba,Sr)SO_4_ Solid Solutions. Minerals 2020, 10, 812. https://doi.org/10.3390/min10090812
